# *In vitro* culture of ovine embryos up to early gastrulating stages

**DOI:** 10.1242/dev.199743

**Published:** 2022-03-24

**Authors:** Priscila Ramos-Ibeas, Leopoldo González-Brusi, María Torres Used, María Jesús Cocero, Pilar Marigorta, Ramiro Alberio, Pablo Bermejo-Álvarez

**Affiliations:** 1 Animal Reproduction Department, INIA-CSIC, Madrid 28040, Spain; 2 School of Biosciences, University of Nottingham, Sutton Bonington Campus, LE12 5RD, UK

**Keywords:** Embryo, *In vitro*, Post-hatching culture, Ovine, Gastrulation

## Abstract

Developmental failures occurring shortly after blastocyst hatching from the zona pellucida constitute a major cause of pregnancy losses in both humans and farm ungulates. The developmental events occurring following hatching in ungulates include the proliferation and maturation of extra-embryonic membranes – trophoblast and hypoblast – and the formation of a flat embryonic disc, similar to that found in humans, which initiates gastrulation prior to implantation. Unfortunately, our understanding of these key processes for embryo survival is limited because current culture systems cannot sustain ungulate embryo development beyond hatching*.* Here, we report a culture system that recapitulates most developmental landmarks of gastrulating ovine embryos: trophoblast maturation, hypoblast migration, embryonic disc formation, disappearance of the Rauber's layer, epiblast polarization and mesoderm differentiation. Our system represents a highly valuable platform for exploring the cell differentiation, proliferation and migration processes governing gastrulation in a flat embryonic disc and for understanding pregnancy failures during the second week of gestation.

This article has an associated ‘The people behind the papers’ interview.

## Abstract (precis)

**Summary:** The development of an *in vitro* post-hatching culture system that sustains most developmental landmarks of sheep embryos up to gastrulating stages is presented, representing an unprecedented advance in ungulate species.

## INTRODUCTION

Developmental failures during the peri-gastrulation period are a major cause of infertility in humans and farm animals, entailing significant economic and social consequences. Understanding the cellular and molecular mechanisms that operate during this period is crucial to overcome pregnancy losses, the majority of which occur during the first weeks of development (Diskin and Morris, 2008; Macklon et al., 2002). Ungulate embryos are routinely produced *in vitro* without the need of experimental animals, using oocytes recovered from ovaries of animals destined for human consumption. Unfortunately, current *in vitro* systems are unable to support embryo development beyond blastocyst hatching, hampering the study of peri-gastrulation stages because of the requirement of experimental animals.

After hatching from the zona pellucida, the mammalian blastocyst is formed by three different lineages: epiblast, which will form the foetus, and hypoblast and trophectoderm (TE), which will develop the foetal part of the placenta (Maddox-Hyttel et al., 2003). In ungulates, the hypoblast migrates to cover the entire inner embryo surface and the epiblast forms a flat embryonic disc (ED) (van Leeuwen et al., 2015), resembling that found in humans and in striking contrast to the egg cylinder developed in mouse embryos (Shahbazi and Zernicka-Goetz, 2018). The hypoblast and the TE undergo extensive growth towards the end of the second week of development, and the embryo transforms from spherical to ovoid, tubular and then filamentous shape before implantation (Artus et al., 2020). Simultaneously, the polar TE covering the epiblast (termed Rauber's layer) disappears and the epiblast forms a polarized epithelium that will initiate gastrulation (van Leeuwen et al., 2020).

Pioneer studies in the 1970s and 1980s reported a certain degree of post-blastocyst development *in vitro* in the mouse model (Gonda and Hsu, 1980; Hsu, 1979; Spindle, 1980; Wiley and Pedersen, 1977), but a highly replicable system able to develop mouse embryos to peri-gastrulating stages has not been available until recently (Bedzhov et al., 2014). The system developed in mouse embryos was later adapted to achieve the development of human embryos up to peri-gastrulating stages equivalent to 14 days post-fertilization (Deglincerti et al., 2016; Shahbazi et al., 2016). In humans, this system has been used to elucidate molecular and morphogenetic events occurring during this period (Xiang et al., 2020; Zhou et al., 2019), known as ‘the black box’ of development (Macklon et al., 2002), and to explore the post-implantation developmental consequences of aneuploidy (Shahbazi et al., 2020). The development of an analogous system in ungulates offers a double benefit. First, it would enable the development of technologies for improving reproductive efficiency in farm animals, in which developmental arrest during this period constitutes the major cause for reproductive failures (Diskin and Morris, 2008). Second, as ungulate gastrulation occurs in a flat ED and genome editing is already available for these species (Lamas-Toranzo et al., 2019), ungulates could emerge as a relevant model to advance our knowledge of human peri-gastrulation events.

Pioneer *in vitro* systems developed in ungulates relied on agarose tunnels and serum- and glucose-enriched medium to achieve certain proliferation of trophoblast and hypoblast in bovine embryos, but failed to support epiblast development (Alexopoulos et al., 2005; Brandão et al., 2004; Vajta et al., 2004). We have recently established an *in vitro* system based on N2B27 medium that supports trophoblast proliferation, hypoblast migration along the entire inner embryo surface and epiblast development into an ED-like structure in bovine embryos (Ramos-Ibeas et al., 2020). However, by day (D) 15 a region of these ED-like structures had lost the expression of the epiblast marker SOX2, suggesting that ED requirements to progress in development and start gastrulation were still not fully fulfilled *in vitro*. Here, we report a culture system based on N2B27 medium supplemented with activin A and Rho-associated protein kinase (ROCK) inhibitor (ROCKi) that allows D14 *in vitro* sheep embryos to recapitulate most developmental landmarks of *in vivo* embryos during the second week of development, including the initiation of gastrulation.

## RESULTS

### Post-hatching sheep development in different culture media

**Fig. 1.**
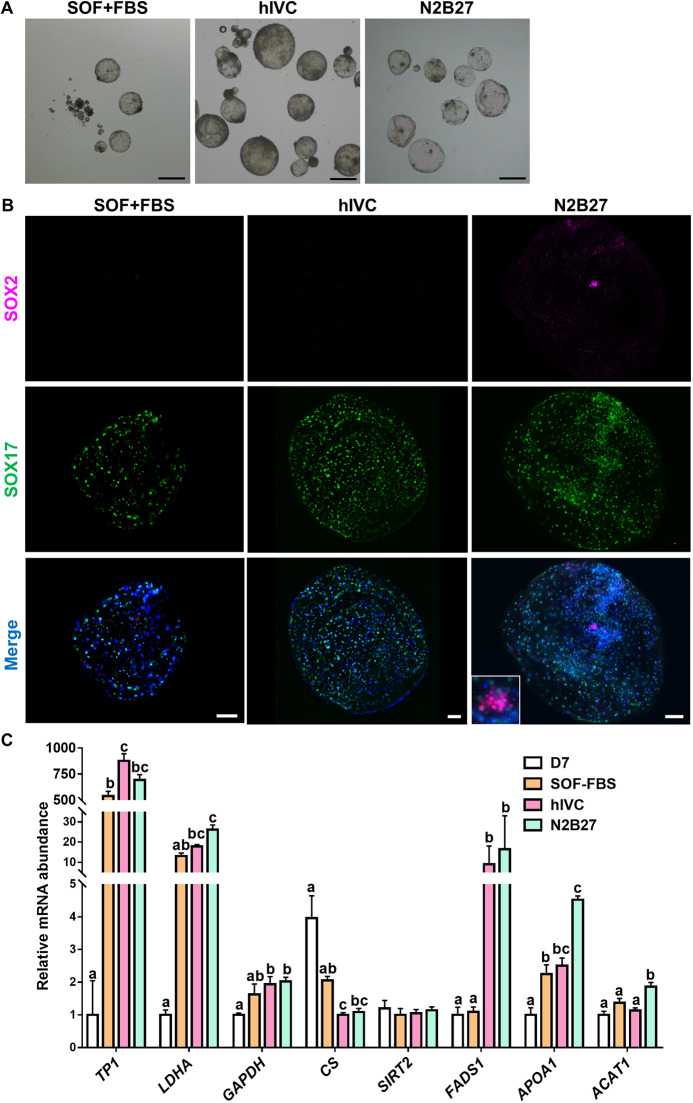
**Post-hatching development *in vitro* in basal media.** (A) Representative brightfield images of D14 embryos cultured in SOF+FBS, hIVC or N2B27. (B) Complete hypoblast migration is achieved in most D14 embryos, but epiblast development is impaired in SOF+FBS and hIVC media. Staining for SOX2 (epiblast) and SOX17 (hypoblast); inset shows magnification of the epiblast. Scale bars: 1 mm (A); 100 µm (B). (C) Relative mRNA abundance in D7 and D14 embryos cultured in SOF+FBS, hIVC or N2B27. Different letters indicate significant differences (one-way ANOVA; *P*<0.05). Bars represent mean±s.e.m.

In contrast to previous observations in bovine blastocysts (Ramos-Ibeas et al., 2020), sheep blastocysts attach to the culture dish surface right after hatching from the zona pellucida. To prevent embryo attachment and subsequent two-dimensional growth, culture dishes were coated with agarose gel. D6/7 *in vitro*-produced blastocysts were randomly allocated to three different culture media that were replaced every other day: (1) SOF (a commonly used medium to develop bovine and ovine embryos; Holm et al., 1999) supplemented with 10% foetal bovine serum (FBS) (hereafter termed SOF+FBS); (2) an *in vitro* culture medium supporting post-implantation development of murine and human embryos (hIVC) (Bedzhov et al., 2014; Deglincerti et al., 2016; Shahbazi et al., 2016); and (3) chemically defined N2B27 medium supporting bovine post-hatching development *in vitro* (Ramos-Ibeas et al., 2020). At D14, collected embryos presented a spherical shape in all conditions, but those cultured in SOF+FBS showed significantly reduced survival and growth compared with the other groups (Table 1, Fig. 1A). In ungulates, hypoblast migrates to cover the inner surface of the trophoblast. Hypoblast (SOX17^+^) migration along the inner surface of the trophoblast was complete in most embryos cultured in hIVC and N2B27, but no epiblast cells (SOX2^+^) were detected at D14 in embryos cultured in SOF+FBS or in hIVC, whereas SOX2^+^ cells were observed in 39.3% of the embryos cultured in N2B27 (Table 1, Fig. 1B).

**Table 1.**
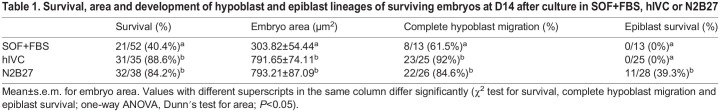
Survival, area and development of hypoblast and epiblast lineages of surviving embryos at D14 after culture in SOF+FBS, hIVC or N2B27

Transcription of trophoblast protein-1 (*TP1*, also known as interferon tau), responsible for maternal recognition of pregnancy in sheep (Godkin et al., 1982), increased at D14 in all culture media, reflecting trophoblast proliferation and development. Transcriptional analysis of rate-limiting enzymes involved in anaerobic glycolysis [lactate dehydrogenase (*LDHA*) and glyceraldehyde-3-phosphate dehydrogenase (*GAPDH*); Granchi et al., 2010], Kreb's cycle [citrate synthase (*CS*); Seedorf et al., 1986] and the pentose phosphate pathway [sirtuin 2 (*SIRT2*); Wang et al., 2014] revealed a metabolic switch from oxidative phosphorylation to anaerobic glycolysis between D7 and D14 of *in vitro* culture. *LDHA* and *GAPDH* were upregulated and *CS* was downregulated at D14 after culture in hIVC and N2B27. Furthermore, fatty acid desaturase 1 (*FADS1*), which increases following *in vivo* elongation (Moraes et al., 2018; Ribeiro et al., 2016), was significantly upregulated after culture in hIVC and N2B27, and acetyl CoA transferase (*ACAT1*) and apolipoprotein A1 (*APOA1*), involved in lipid storage (Schultz et al., 2019; Tian et al., 2012), were significantly increased after culture in N2B27 medium (Fig. 1C).

### Epiblast development *in vitro* is promoted by activin A and ROCKi

**Fig. 2.**
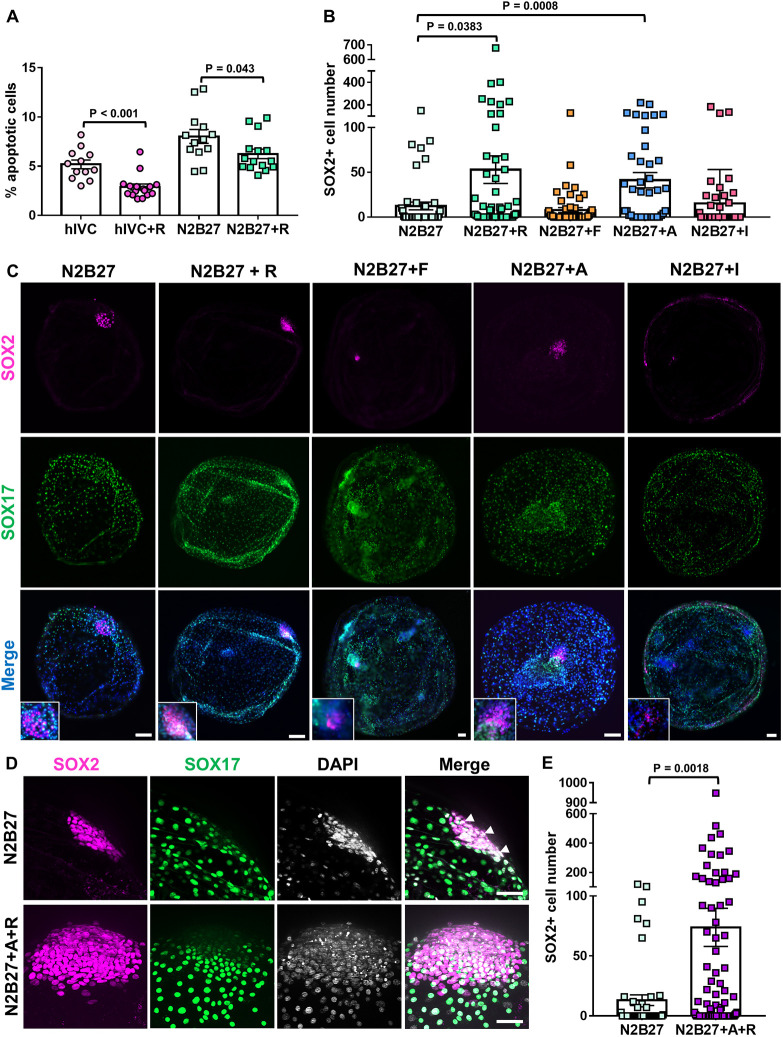
**Epiblast development is improved by activin A and ROCKi supplementation.** (A) ROCKi significantly reduced apoptosis in embryos cultured in hIVC. hIVC, *n*=12; hIVC+R, *n*=16; N2B27, *n*=13; N2B27+R, *n*=15; Mann–Whitney Rank Sum test. (B) SOX2^+^ epiblast cells of embryos cultured in N2B27 (*n*=49), N2B27+R (*n*=79), N2B27+F (*n*=51), N2B27+A (*n*=46) or N2B27+I (*n*=46); one-way ANOVA, Kruskal–Wallis test. (C) Representative embryos stained for SOX2 (epiblast) and SOX17 (hypoblast). Insets show magnifications of the epiblast. (D) Representative EDs with (N2B27) and without (N2B27+A+R) Rauber's layer stained for SOX2 (epiblast) and SOX17 (hypoblast). Arrowheads indicate trophoblast cells covering the epiblast in embryos cultured in N2B27. (E) SOX2^+^ epiblast cells of embryos cultured in N2B27 (*n*=48) or N2B27+A+R (*n*=88); Mann–Whitney Rank Sum test. A, 20 ng/ml activin A; I, 100 ng/ml IGF1; F, 20 ng/ml bFGF; R, ROCK inhibitor (10 μM Y-27632). Bars represent mean±s.e.m. Scale bars: 100 µm (C); 50 µm (D).

Given that epiblast survival was the most limiting factor for proper post-hatching development *in vitro*, we tested whether ROCK inhibition could promote epiblast survival in hIVC, as recently reported for human embryos (Xiang et al., 2020). ROCKi (Y-27632) supplementation to hIVC medium (hIVC+R) reduced significantly the percentage of apoptotic cells in cultured embryos (Fig. 2A, Fig. S1A), but there was no positive effect on embryo survival and size, or on hypoblast migration, and epiblast cells were still not detected at D14 in hIVC+R (Table S1, Fig. S1B,C). However, few SOX2^+^ cells (4.6±1.3/embryo) were found in D11 embryos cultured in hIVC+R, revealing that epiblast cells were gradually lost during culture in these conditions.

Aiming to further promote epiblast development in N2B27, the only medium able to support epiblast survival up to D14, we supplemented N2B27 with different molecules: ROCKi (Xiang et al., 2020) (N2B27+R); basic fibroblast growth factor (bFGF), which promotes epiblast pluripotency *in vitro* in bovine embryonic stem cells (Bogliotti et al., 2018) (N2B27+F); activin A, which activates the TFGβ pathway and is involved in epiblast development (Ramos-Ibeas et al., 2019) (N2B27+A); and IGF1, which is expressed in the epiblast (Ramos-Ibeas et al., 2019) and improves human embryo and epiblast survival *in vitro* (Spanos et al., 2000) (N2B27+I). Activin A and ROCKi supplementation significantly increased the percentage of embryos with surviving epiblast (Table 2) and promoted the proliferation of SOX2^+^ cells (41.0±8.6 and 52.9±15.4 cells/embryo, respectively, versus 12.3±4.3 in N2B27 alone; mean±s.e.m.; Fig. 2C). bFGF and IGF1 supplementation did not improve any of the parameters analysed, and IGF1 caused a significant reduction (6.7 versus 40%) in the percentage of embryos showing a compact ED composed of >50 SOX2^+^ cells (Table 2, Fig. 2C).

**Table 2.**
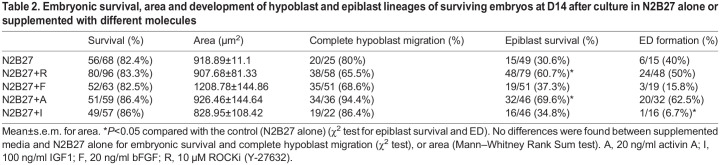
Embryonic survival, area and development of hypoblast and epiblast lineages of surviving embryos at D14 after culture in N2B27 alone or supplemented with different molecules

Next, we tested a combination of the molecules that yielded the best results in terms of epiblast development: ROCKi and activin A (N2B27+A+R). Epiblast survival and SOX2^+^ cell number were significantly enhanced in N2B27+A+R compared with N2B27 alone (73.7±15.9 versus 13.1±4.4 cells/embryo; mean±s.e.m.). The percentage of embryos showing an ED at D14 was also significantly increased in N2B27+A+R (Table 3, Fig. 2D,E). Moreover, disintegration of Rauber's layer (the polar trophoblast, disappearing in ungulates through apoptosis; van Leeuwen et al., 2020) was detected in 22 out of 36 (61.1%) EDs developed in N2B27+A+R (Fig. 2D, Fig. S2), whereas this layer was present in all EDs developed in N2B27 alone (Fig. 2D, Fig. S3).

**Table 3.**
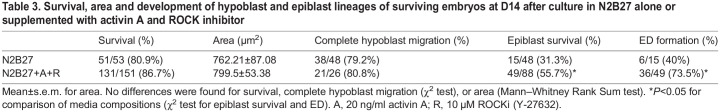
Survival, area and development of hypoblast and epiblast lineages of surviving embryos at D14 after culture in N2B27 alone or supplemented with activin A and ROCK inhibitor

### Characterization of *in vitro* developed embryos

**Fig. 3.**
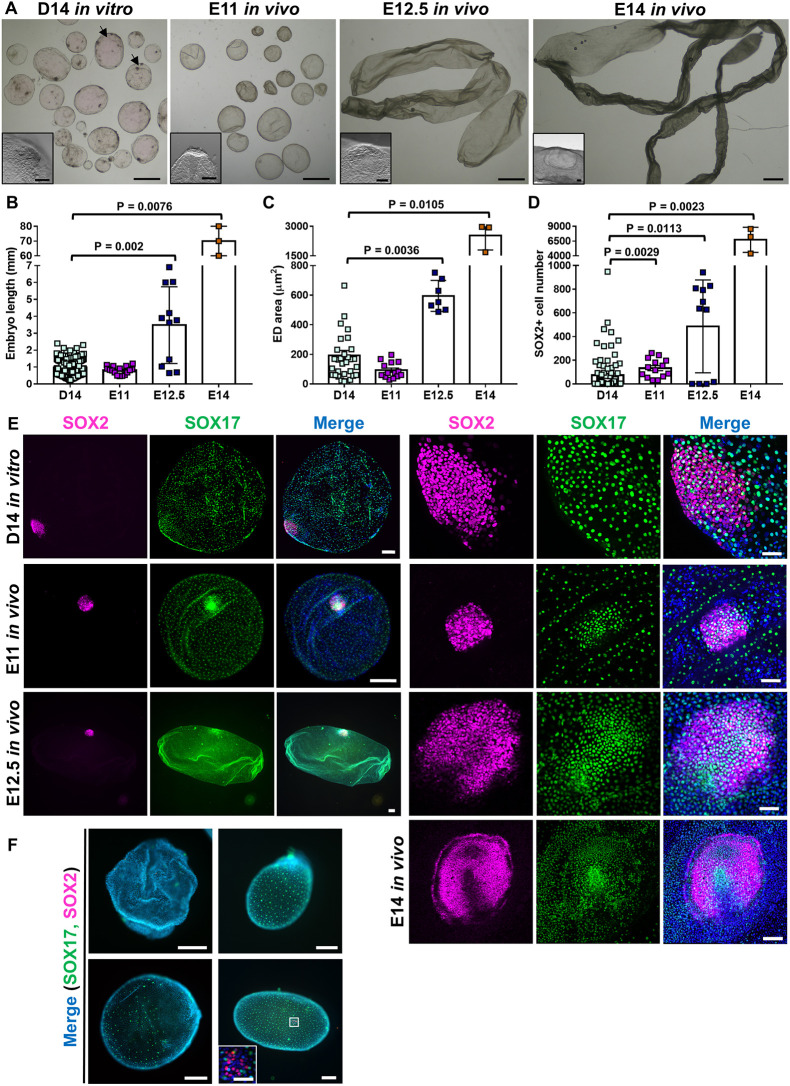
**Comparison between *in vitro* and *in vivo* post-hatching embryos.** (A) Representative *in vitro-*produced D14 embryos cultured in N2B27+A+R and E11, E12.5 and E14 *in vivo-*derived embryos. Insets show magnifications of the ED. Arrows point to *in vitro* embryos with several dark areas. (B) Embryo length (mm) of *in vitro-*produced D14 embryos cultured in N2B27+A+R (*n*=131) and E11 (*n*=16), E12.5 (*n*=11) and E14 (*n*=3) *in vivo-*derived embryos. (C) ED area (µm^2^) of *in vitro-*produced D14 embryos cultured in N2B27+A+R (*n*=32) and E11 (*n*=14), E12.5 (*n*=7) and E14 (*n*=3) *in vivo-*derived embryos. (D) SOX2^+^ epiblast cells in *in vitro-*produced D14 embryos cultured in N2B27+A+R (*n*=88) and E11 (*n*=14), E12.5 (*n*=11) and E14 (*n*=3) *in vivo-*derived embryos. Bars represent mean±s.e.m.; one-way ANOVA, Kruskal–Wallis test. (E) Representative *in vitro-*produced D14 embryo cultured in N2B27+A+R and E11 and E12.5 *in vivo-*derived embryos. Panels on the right show magnifications of EDs from D14 *in vitro* and E11, E12.5 and E14 *in vivo* embryos. Staining for SOX2 (epiblast) and SOX17 (hypoblast). (F) E12.5 *in vivo*-derived embryos showing developmental arrest or delay, stained for SOX2 (epiblast) and SOX17 (hypoblast). Inset shows sparse epiblast cells. Scale bars: 1 mm (A); 100 µm (insets in A); 200 µm for E (left) and F; 100 µm for E14 ED in F (right); 50 µm for D14, E11 and E12.5 EDs in E (right) and for magnification in F.

The developmental landmarks achieved by D14 *in vitro* embryos cultured in N2B27+A+R were compared with those attained by *in vivo* embryos collected at days 11, 12.5 and 14 post-mating [embryonic day (E) 11, E12.5 and E14]. D14 *in vitro* embryos remained spherical, similar to E11 *in vivo* embryos, with an obvious ED protruding at the surface of the most advanced embryos. However, *in vitro* embryos appeared more translucent and frequently showed dark areas (Fig. 3A). At E12.5, most *in vivo* embryos already showed an ovoid shape (*n*=8/11; 72.7%) and at E14 all recovered embryos were tubular (*n*=3/3)*.* Embryo length and ED area of D14 *in vitro* embryos were significantly smaller than *in vivo* embryos at E12.5 and E14 (Fig. 3B,C).

Hypoblast migration along the entire inner trophoblast surface was completed in 80.8% of D14 *in vitro* embryos (*n*=21/26), and in 92.8% of E11 (*n*=13/14) and 81.8% of E12.5 (*n*=9/11) *in vivo* embryos (Fig. 3E). The number of SOX2^+^ epiblast cells at D14 *in vitro* and E11 and 12.5 *in vivo* was very variable within each group (Fig. 3D). The high variability in E12.5 *in vivo* embryos was associated with certain developmental arrest in 4/11 embryos, which lacked an ED (Fig. 3F). The Rauber's layer was absent in 22/36 D14 *in vitro* embryos containing an ED (61.1%; Fig. 2D, Fig. S2), whereas in E11 *in vivo* embryos it was absent in 1/5 (20%), disappearing in 1/5 (20%) and present in 3/5 (60%) embryos (Fig. S4A-D). By E12.5 *in vivo*, Rauber's layer was absent in all embryos containing an ED (*n*=7/7).

**Fig. 4.**
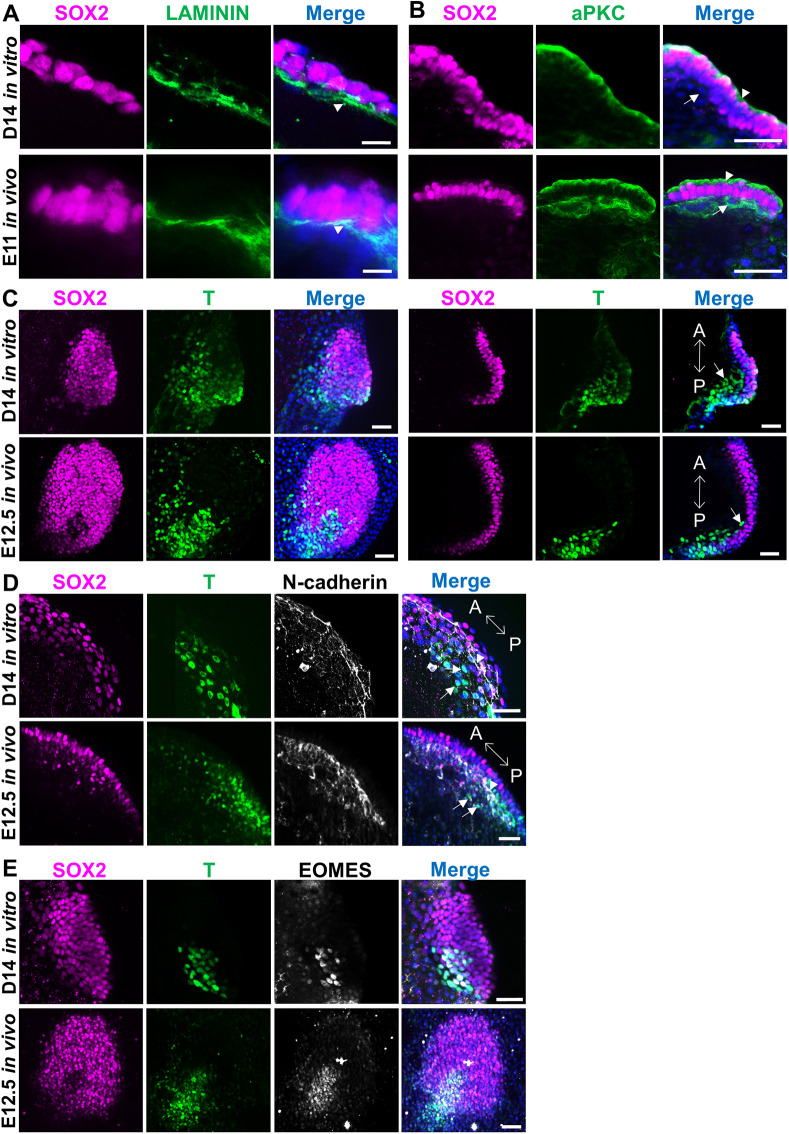
**Epiblast and mesoderm development in *in vitro* and *in vivo* post-hatching embryos.** (A) Basement membrane formation under the epiblast in D14 *in vitro* and E11 *in vivo* embryos. Laminin accumulation can be seen on the basal side of SOX2^+^ epiblast cells (arrowheads). Maximum projections (*z*-sections 6-8 in Fig. S5A and 7 and 8 in Fig. S5B). (B) Polarization of SOX2^+^ epiblast cells in D14 *in vitro* and E11 *in vivo* embryos revealed by apical localization of aPKC (arrowheads). Arrow points to hypoblast cells. Maximum projections (*z*-sections 4 and 5 in Fig. S5C and 4 and 5 in Fig. S5D). (C) EDs in a D14 *in vitro* and in an E12.5 *in vivo* embryo initiating gastrulation and showing mesoderm cells in the posterior part, stained for T. Images on the left are maximum projections (*z*-sections 1-16 in Fig. S6A and 1-21 in Fig. S6B). Images on the right show a section of the intermediate part of the structure. Double arrow indicates anterior-posterior (A-P) axis. Arrows point to migrating mesoderm cells. (D) EDs in a D14 *in vitro* and in an E12.5 *in vivo* embryo showing mesoderm cells stained for T and expression of the EMT marker N-cadherin. Maximum projections (*z*-sections 1-11 in Fig. S7A and 1-8 in Fig. S7B). Arrows point to migrating mesoderm cells, arrowheads point to N-cadherin-positive cells and double arrow indicates A-P axis. (E) EDs in a D14 *in vitro* and in an E12.5 *in vivo* embryo showing mesoderm cells stained for T and EOMES; maximum projections. Scale bars: 10 µm (A); 50 µm (B-E).

The formation of a basement membrane, required for the polarization of epiblast cells and identifiable by the presence of laminin in the basal side of epiblast cells (Bedzhov and Zernicka-Goetz, 2014; Oestrup et al., 2009; Xiang et al., 2020), was detected from E11 *in vivo* (*n*=2/2) and in 77.8% D14 *in vitro* embryos with ED (*n*=7/9) (Fig. 4A, Fig. S5A,B). Accordingly, epiblast cells were apico-basally polarized in E11 *in vivo* (*n*=3/3) and D14 *in vitro* embryos with ED (*n*=4/4), as determined by apical localization of aPKC, the principal kinase of the apical Par polarity complex (Goldstein and Macara, 2007; Shahbazi et al., 2016) (Fig. 4B, Fig. S5C,D).

Gastrulation remains poorly defined in ungulates because it can only be investigated in elongated embryos recovered *in vivo*, with an elevated effort and economic cost. The first hallmark of primitive streak formation is the expression of T-box transcription factor (T or brachyury), which can be detected in bovine and ovine embryos in the posterior part of the ED (Guillomot et al., 2004; van Leeuwen et al., 2015). T^+^ mesoderm cells were detected in 41.2% D14 *in vitro* embryos containing an ED (*n*=14/34). Most T^+^ cells had repressed SOX2 expression and were located at the posterior part of the ED, denoting the initiation of gastrulation and symmetry breaking. Moreover, some T^+^ cells had already started migration to cover the inner surface of the ED (Fig. 4C, Fig. S6A), and had initiated epithelial-mesenchymal transition (EMT), showing N-cadherin upregulation in 44.4% D14 *in vitro* embryos containing an ED (*n*=4/9) (Fig. 4D, Fig. S7A). No T expression was detected in E11 *in vivo* embryos (*n*=8), but T^+^ cells were detected in all E12.5 embryos analysed (*n*=14), some of which were already delaminating along the inner surface of the ED (Fig. 4C, Fig. S6B) and expressing the EMT marker N-cadherin (*n*=5/5) (Fig. 4D, Fig. S7B). Another T-box protein playing a major role in primitive streak formation and mesoderm patterning is eomesodermin (EOMES), which is expressed together with T in the posterior part of the ED in the sheep (Guillomot et al., 2004). T^+^/EOMES^+^ cells were detected in D14 *in vivo* (*n*=3/3) and E12.5 *in vivo* (*n*=3/3) embryos (Fig. 4E).

**Fig. 5.**
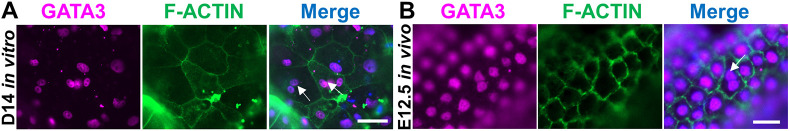
**Trophoblast development in *in vitro* and *in vivo* post-hatching embryos.** (A,B) Binucleate trophoblast cells are indicated by arrows in a D14 *in vitro* embryo (A) and an E12.5 *in vivo* embryo (B). GATA3 (trophoblast) and F-actin (cellular membranes) staining. Scale bars: 20 µm.

During embryo elongation in ungulates, some trophoblast cells differentiate by nuclear division without cytokinesis to form binucleate cells (BNCs), which will fuse with endometrial epithelial cells to form trinucleate cells after implantation (Hoffman and Wooding, 1993; Wimsatt, 1951). *In vitro*, BNCs were identified at D14 by nuclear staining with GATA3 (Gerri et al., 2020) and membrane labelling with F-actin (*n*=8/15, 53.3%; Fig. 5A), but they were still not present in D12 embryos (*n*=23). *In vivo*, BNCs were not observed at E11 (*n*=3), but they were present in E12.5 embryos (*n*=3) (Fig. 5B). Trophoblast cells in D14 *in vitro* embryos were more scattered and had a larger cytoplasm than in E12.5 *in vivo* embryos, which could be a result of a lower proliferation rate and the failure to switch from spherical to ovoid shape *in vitro.*

**Fig. 6.**
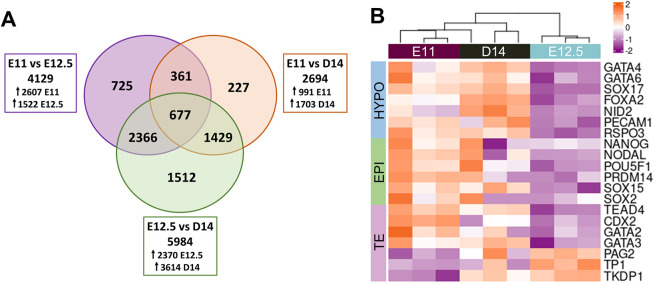
**Transcriptional analysis of *in vitro* and *in vivo* post-hatching embryos by RNA-seq.** (A) Venn diagram for DEGs identified for E11 versus E12.5 *in vivo*; E11 *in vivo* versus D14 *in vitro*; and E12.5 *in vivo* versus D14 *in vitro* (shrunken FC>2; *P*adj<0.01). (B) Heatmap of expression levels of selected lineage-specific genes (log2-normalized gene counts). EPI, epiblast; HYPO, hypoblast; TE, trophectoderm.

Finally, to further contrast *in vitro-*developed embryos with their *in vivo* counterparts at the two closest developmental stages, a comparative transcriptomic analysis was performed by RNA sequencing (RNA-seq) on D14 *in vitro*, and E11 and E12.5 *in vivo* embryos. Analysis of differentially expressed genes (DEGs) (log2 fold change>2, *P*<0.01) identified 2694 DEGs between D14 *in vitro* and E11 *in vivo* embryos, 5984 DEGs between D14 *in vitro* and E12.5 *in vivo* embryos and 4129 DEGs between E11 and E12.5 *in vivo* embryos from a total of 14,359 expressed genes (Fig. 6A, Table S2). The transcriptional differences observed for known lineage markers suggested that D14 embryos were transcriptionally closer to E11 *in vivo* embryos. Hypoblast- and epiblast-specific genes were upregulated in E11 *in vivo* and in D14 *in vitro* compared with E12.5 *in vivo* embryos. Similarly, early TE markers (*TEAD4*, *CDX2*, *GATA2* and *GATA3*) were highly expressed in E11 *in vivo* and in D14 *in vitro* embryos, whereas late TE markers (*PAG2*, *TP1* and *TKDP1*) were upregulated in E12.5 *in vivo* embryos (Fig. 6B).

## DISCUSSION

Here, we report a system to extend the current *in vitro* culture end-point from hatched sheep blastocysts up to gastrulating stages. By providing a non-adherent surface and N2B27 medium supplemented with activin A and ROCKi, embryos can recapitulate *in vitro* most developmental processes of *in vivo* embryos, including trophoblast proliferation and differentiation, hypoblast migration along the entire inner embryo surface, ED formation, shedding of Rauber's layer, epiblast polarization, and onset of mesoderm specification and migration. Embryos fully developed *in vitro* reached a developmental stage that resembles E11 (spherical shape and similar transcriptional profile) or E12.5 (epiblast polarization, onset of mesoderm specification and migration and shedding of Rauber's layer) *in vivo* embryos. These developmental landmarks were achieved following 14 days of culture, evidencing an accumulated developmental delay of 1.5-3 days compared with *in vivo* development. Although maternal histotroph seems still necessary to support further development of the embryo, our system reveals a remarkable self-organizing capacity of sheep embryos in the absence of maternal influence, resembling early gastrulating stages.

So far, the absence of a system to support *in vitro* development beyond the blastocyst stage has limited our understanding of embryogenesis after blastocyst hatching and early pregnancy failure in ungulates. Previous studies have reported post-hatching survival and growth of bovine embryos *in vitro* inside agarose tunnels in conventional pre-hatching embryo culture medium (SOF) supplemented with glucose and FBS. However, under these conditions only trophoblast and hypoblast, to a lesser degree, showed successful proliferation (Brandão et al., 2004; Vajta et al., 2004). We have previously observed that agarose tunnels do not provide any developmental advantage to *in vitro* post-blastocyst development in bovine embryos and that epiblast survival is not supported in this medium (Ramos-Ibeas et al., 2020). In agreement with these reports, post-blastocyst culture in SOF+FBS yielded limited embryo survival and growth in ovine embryos, with reduced hypoblast proliferation and no epiblast survival. Successful post-blastocyst culture and epiblast development has been achieved in mouse and human embryos cultured in IVC medium (Bedzhov et al., 2014; Deglincerti et al., 2016; Shahbazi et al., 2016), but sheep embryos failed to maintain their epiblast when cultured in this medium, even when it was supplemented with ROCKi (hIVC; Xiang et al., 2020). This difference could be explained by species-specific differences in the metabolic requirements of the developing embryo. Lipid compounds present in N2B27 medium (Ramos-Ibeas et al., 2020) and absent in hIVC may be required for ungulate embryo development, as uterine fluid lipidome seems to play a relevant role in elongation (Simintiras et al., 2019), whereas the presence of serum replacement (KSR) in hIVC may be detrimental for embryo development. In agreement with this hypothesis, increased expression of key genes related to lipid metabolism, previously associated with post-hatching embryo development (Moraes et al., 2018; Ribeiro et al., 2016), was mainly observed after culture in N2B27, and embryos cultured in hIVC showed a darker appearance than those developed in other culture media, which might be due to the presence of KSR, previously attributed to detrimental accumulation of lipid droplets in bovine embryos (Brinkhof et al., 2017). Similarly, a metabolic switch from oxidative phosphorylation to anaerobic glycolysis is key to produce crucial metabolites for embryo development after blastocyst hatching (Krisher and Prather, 2012). Increased expression of rate-limiting enzymes involved in anaerobic glycolysis, *LDHA* and *GAPDH*, together with reduced expression of the Kreb's cycle enzyme *CS*, suggested that this metabolic switch takes place also under *in vitro* conditions, particularly in N2B27 medium (Fig. 1C), as we previously observed in bovine embryos (Ramos-Ibeas et al., 2020).

Also in agreement with previous observations in the bovine model (Ramos-Ibeas et al., 2020), N2B27 supported complete hypoblast migration and functional development of the trophoblast, which expressed the trophoblast marker GATA3 (Negrón-Pérez et al., 2017; Ramos-Ibeas et al., 2019) and *TP1*, the major pregnancy recognition signal in ovine (Godkin et al., 1982). Furthermore, BNCs were detected in half of the D14 *in vitro* embryos analysed. These cells produce proteins that are required for placental development (Hashizume et al., 2007) and emerge at a very low proportion in sheep embryos between E12 and E16 (Carnegie et al., 1985), accounting for 15-20% of the trophoblast cells by the time of implantation (Wooding, 1982). Accordingly, we did not find BNCs until E12.5 in *in vivo* sheep embryos. Nevertheless, trophoblast cells showed a slower proliferation rate *in vitro* than in *in vivo* embryos, failing to shape the embryo from spherical to ovoid. Notably the impaired TE development did not interfere with the developmental progression of the epiblast in *in vitro* embryos.

Epiblast was found to be the most sensitive lineage following both *in vitro* and *in vivo* development. *In vitro*, 49/88 (55.7%) embryos showed surviving epiblast cells after culture in N2B27+A+R, although with a high variability in SOX2^+^ cell number, and only 36 of them (40.9% from the total) had formed an ED. Similarly, *in vivo* only 7/11 (63.6%) E12.5 embryos showed an ED, and a high variability in SOX2^+^ cell number was already detected at E11 (ranging from 30 to 261 cells), with 6/14 (42.8%) embryos showing significantly fewer SOX2^+^ cells than their counterparts (<90 cells). These differences were not observed at E14, presumably because those embryos lacking an ED had already degenerated. These observations suggest that failures in epiblast development may underlie the intrinsic high embryo mortality reported during this period of development in both humans and ungulates (Diskin and Morris, 2008; Macklon et al., 2002; Perez-Gomez et al., 2021). N2B27 was the only medium supporting epiblast survival and ED formation in *in vitro*-cultured sheep embryos. This medium has been reported to increase blastocyst cell number during pig and bovine *in vitro* pre-hatching embryo culture (Brinkhof et al., 2017; Rodriguez et al., 2012), and to support the formation of an ED-like structure in D15 bovine embryos, although with partial loss of SOX2 expression (Ramos-Ibeas et al., 2020). N2B27 supplementation with activin A and ROCKi further increased the percentage of embryos with a surviving epiblast and high SOX2^+^ cell number. The activin/TGFβ pathway is essential for epiblast development in pig and human embryos (Alberio et al., 2010; Blakeley et al., 2015; Ramos-Ibeas et al., 2019) and ROCKi has been previously added to IVC medium to increase the percentage of embryos that progress in development until day 14 in humans, achieving ED formation (Xiang et al., 2020).

Combined supplementation with ROCKi and activin A supported key developmental processes in the ED. Shedding of the Rauber's layer was detected in ∼61% of the D14 embryos with an ED cultured in N2B27+A+R, in contrast to embryos cultured in N2B27 without supplementation, and mimicking the gradual loss observed between E11 and E12.5 *in vivo*, as described for bovine embryos (van Leeuwen et al., 2015). Epiblast development also involves cell polarization and formation of a basement membrane, determined by apical localization of aPKC and laminin accumulation, respectively. These events, previously reported in mouse and human embryos cultured *in vitro* (Bedzhov and Zernicka-Goetz, 2014; Shahbazi et al., 2016; Xiang et al., 2020) but not in ungulates, were also observed in most *in vitro*-cultured D14 embryos, similar to E11 *in vivo* embryos. Following cell polarization, gastrulation constitutes the next major developmental step, as it marks the establishment of the anterio-posterior axis, breaking embryo symmetry, and the formation of the three primary germ layers (Solnica-Krezel and Sepich, 2012). *In vitro* D14 embryos displayed T/EOMES^+^ mesoderm cells with downregulation of the epiblast marker SOX2, and expression of the EMT marker N-cadherin. Some of these cells were starting to migrate to cover the inner surface of the ED. Similar developmental events have been reported in D14 *in vitro* human embryos, which contained T^+^ cells showing downregulation of the epiblast marker OCT4 and migrating toward the endoderm (Xiang et al., 2020). *In vivo*, T^+^ cells were not observed up to E12.5, in agreement with a previous report detecting *T* mRNA expression by *in situ* hybridization in E12-E13 sheep embryos (Guillomot et al., 2004).

Global transcriptional analysis revealed a closer proximity between D14 *in vitro* and E11 *in vivo* embryos, as a higher number of DEGs was detected between D14 *in vitro* and E12.5 *in vivo* than between D14 *in vitro* and E11 *in vivo* embryos. Moreover, E11 *in vivo* embryos seemed transcriptionally closer to D14 *in vitro* than to E12.5 *in vivo* embryos, according to the number of DEGs detected. A specific look at known lineage markers found a higher expression of epiblast, hypoblast and early TE markers on both E11 and D14 embryos compared with E12.5. Although the reduced expression of epiblast markers in E12.5 embryos could be caused by the reduced proportion of epiblast cells (epiblast:total ratio) in E12.5 embryos compared with E11 or D14, the upregulation of both hypoblast and early TE markers suggests an earlier stage of differentiation/maturation of these extra-embryonic lineages in E11 and D14 embryos compared with E12.5. This agrees with the higher expression of genes involved in TE maturation and maternal recognition of pregnancy found in E12.5 versus E11 or D14, and may be responsible for the spherical morphology observed in E11 and D14 embryos as opposed to the tubular E12.5 embryos.

In conclusion, we provide a system that extends the current window of embryo development *in vitro* in sheep. Although additional studies will be necessary to attain later developmental processes, such as further development of extra-embryonic membranes or ED development beyond gastrulation, key developmental processes occurring during the second week of development *in vivo* that could not be recapitulated *in vitro* before in any ungulate species (ED formation, shedding of Rauber's layer, epiblast polarization and initiation of gastrulation) are now available for developmental studies. This system will facilitate the exploration of the mechanisms involved in embryo development and pregnancy failure during the second week of gestation, the most susceptible period for developmental failure in farm ungulates and humans, bypassing the need for experimental animals. Moreover, this system will prolong the developmental window in which the integration of pluripotent stem cells into an embryo can be analysed *in vitro* up to gastrulating stages. Given the similarities between ungulate and human gastrulation and the ethical limitations for human embryo culture beyond primitive streak formation, ungulate embryos could emerge as a relevant model for comparative developmental biology.

## MATERIALS AND METHODS

### *In vitro* embryo production and recovery

*In vitro* embryo production procedures were performed as previously described (Cocero et al., 2019). *In vivo*-derived blastocysts were obtained from superovulated ewes on days 11 and 12.5 after mating. For details, see supplementary Materials and Methods.

### Post-hatching development system

D6/7 blastocysts were transferred to agarose-coated four-well dishes (Ramos-Ibeas et al., 2020) in the different culture media tested. In a first experiment, the following media were used: (1) SOF supplemented with 10% (v/v) FBS; (2) an *in vitro* culture medium supporting post-blastocyst development in human embryos (hIVC) (Deglincerti et al., 2016); and (3) N2B27 medium. In subsequent experiments, the following combinations were used: hIVC alone; hIVC supplemented with 10 μM ROCKi (Y-27632, Stem Cell Technologies); N2B27 alone; or N2B27 supplemented with 10 μM ROCK inhibitor, 20 ng/ml activin A (Stem Cell Technologies), 100 ng/ml insulin growth factor 1 (IGF1, Thermo Fisher Scientific) or 20 ng/ml bFGF (Thermo Fisher Scientific), or a combination of 10 μM ROCKi and 20 ng/ml activin A. For details, see supplementary Materials and Methods.

### Immunofluorescence

Embryos were fixed in 4% paraformaldehyde for 15 min, washed in PBS with 1% bovine serum albumin (BSA), permeabilized in 1% Triton X-100 in PBS for 15 min at room temperature (RT) and blocked in 10% donkey serum and 0.02% Tween 20 in PBS for 1 h at RT. Then, they were incubated overnight at 4°C with primary antibodies (Table S3). After four washes in 1% BSA in PBS, embryos were incubated in the appropriate secondary Alexa-conjugated antibodies (Table S3) and counterstained with DAPI for 1 h at RT, followed by four washes in 1% BSA in PBS. Finally, embryos were mounted and imaged at a Zeiss Axio Observer microscope coupled to ApoTome.2 or a fluorescence stereomicroscope (Zeiss V20). For details, see supplementary Materials and Methods.

### Apoptotic cell detection

The TdT-mediated dUTP-biotin Nick end-labelling (TUNEL) assay was employed for apoptotic cell detection using the In Situ Cell Death Detection Kit, TMR Red (Roche). For details, see supplementary Materials and Methods.

### RNA isolation, cDNA synthesis and qPCR

Poly (A) RNA was extracted from four individual whole D14 embryos of each group and four pools of ten D7 blastocysts using the Dynabeads mRNA Purification Kit (Life Technologies) following the manufacturer's instructions with minor modifications. mRNA transcripts were quantified by real-time quantitative PCR (qPCR) following a previously described protocol. Two replicate PCR experiments were conducted for all genes of interest. Primer sequences are provided in Table S4 and more details are provided in supplementary Materials and Methods.

### RNA sequencing

Total RNA was extracted from three D14 *in vitro*, three E11 and three E12.5 *in vivo* embryos using the MagMAX™ *mir*Vana™ Total RNA Isolation kit. cDNA was synthetized with SMART-Seq™ v4 Ultra™ Low Input RNA Kit (Clontech), libraries were prepared using Covaris shearing system and sequencing on an Illumina system. For details, see supplementary Materials and Methods.

### Data and statistical analysis

Data analysis was manual, blinded and performed by different researchers. Data were analysed using GraphPad Prism (GraphPad Software) and SigmaStat (Systat Software) packages. For details, see supplementary Materials and Methods.

## Supplementary Material

Supplementary information

Reviewer comments

## References

[DEV199743C1] Alberio, R., Croxall, N. and Allegrucci, C. (2010). Pig epiblast stem cells depend on activin/nodal signaling for pluripotency and self-renewal. *Stem Cells Dev.* 19, 1627-1636. 10.1089/scd.2010.0012 20210627 PMC3129689

[DEV199743C2] Alexopoulos, N. I., Vajta, G., Maddox-Hyttel, P., French, A. J. and Trounson, A. O. (2005). Stereomicroscopic and histological examination of bovine embryos following extended in vitro culture. *Reprod. Fertil. Dev.* 17, 799-808. 10.1071/RD04104 16476207

[DEV199743C3] Artus, J., Hue, I. and Acloque, H. (2020). Preimplantation development in ungulates: a ‘ménage à quatre’ scenario. *Reproduction* 159, R151-R172. 10.1530/REP-19-0348 31751293

[DEV199743C4] Bedzhov, I. and Zernicka-Goetz, M. (2014). Self-organizing properties of mouse pluripotent cells initiate morphogenesis upon implantation. *Cell* 156, 1032-1044. 10.1016/j.cell.2014.01.023 24529478 PMC3991392

[DEV199743C5] Bedzhov, I., Leung, C. Y., Bialecka, M. and Zernicka-Goetz, M. (2014). In vitro culture of mouse blastocysts beyond the implantation stages. *Nat. Protoc.* 9, 2732-2739. 10.1038/nprot.2014.186 25356584

[DEV199743C6] Blakeley, P., Fogarty, N. M. E., del Valle, I., Wamaitha, S. E., Hu, T. X., Elder, K., Snell, P., Christie, L., Robson, P. and Niakan, K. K. (2015). Defining the three cell lineages of the human blastocyst by single-cell RNA-seq. *Development* 142, 3151-3165. 10.1242/dev.131235 26293300 PMC4582176

[DEV199743C7] Bogliotti, Y. S., Wu, J., Vilarino, M., Okamura, D., Soto, D. A., Zhong, C., Sakurai, M., Sampaio, R. V., Suzuki, K., Izpisua Belmonte, J. C. et al. (2018). Efficient derivation of stable primed pluripotent embryonic stem cells from bovine blastocysts. *Proc. Natl. Acad. Sci. USA* 115, 2090-2095. 10.1073/pnas.1716161115 29440377 PMC5834688

[DEV199743C8] Brandão, D. O., Maddox-Hyttel, P., Løvendahl, P., Rumpf, R., Stringfellow, D. and Callesen, H. (2004). Post hatching development: a novel system for extended in vitro culture of bovine embryos. *Biol. Reprod.* 71, 2048-2055. 10.1095/biolreprod.103.025916 15329327

[DEV199743C9] Brinkhof, B., van Tol, H. T. A., Groot Koerkamp, M. J., Wubbolts, R. W., Haagsman, H. P. and Roelen, B. A. J. (2017). Characterization of bovine embryos cultured under conditions appropriate for sustaining human naïve pluripotency. *PLoS ONE* 12, e0172920. 10.1371/journal.pone.0172920 28241084 PMC5328396

[DEV199743C11] Carnegie, J. A., McCully, M. E. and Robertson, H. A. (1985). The early development of the sheep trophoblast and the involvement of cell death. *Am. J. Anat.* 174, 471-488. 10.1002/aja.1001740409 4083261

[DEV199743C12] Cocero, M. J., Marigorta, P., Novillo, F., Folch, J., Sánchez, P., Alabart, J. L. and Lahoz, B. (2019). Ovine oocytes display a similar germinal vesicle configuration and global DNA methylation at prepubertal and adult ages. *Theriogenology* 138, 154-163. 10.1016/j.theriogenology.2019.07.011 31357118

[DEV199743C13] Deglincerti, A., Croft, G. F., Pietila, L. N., Zernicka-Goetz, M., Siggia, E. D. and Brivanlou, A. H. (2016). Self-organization of the in vitro attached human embryo. *Nature* 533, 251-254. 10.1038/nature17948 27144363

[DEV199743C14] Diskin, M. G. and Morris, D. G. (2008). Embryonic and early foetal losses in cattle and other ruminants. *Reprod. Domestic Anim.=Zuchthygiene* 43 Suppl. 2, 260-267. 10.1111/j.1439-0531.2008.01171.x 18638133

[DEV199743C15] Gerri, C., McCarthy, A., Alanis-Lobato, G., Demtschenko, A., Bruneau, A., Loubersac, S., Fogarty, N. M. E., Hampshire, D., Elder, K., Snell, P. et al. (2020). Initiation of a conserved trophectoderm program in human, cow and mouse embryos. *Nature* 587, 443-447. 10.1038/s41586-020-2759-x 32968278 PMC7116563

[DEV199743C16] Godkin, J. D., Bazer, F. W., Moffatt, J., Sessions, F. and Roberts, R. M. (1982). Purification and properties of a major, low molecular weight protein released by the trophoblast of sheep blastocysts at day 13-21. *J. Reprod. Fertil.* 65, 141-150. 10.1530/jrf.0.0650141 7077590

[DEV199743C17] Goldstein, B. and Macara, I. G. (2007). The PAR proteins: fundamental players in animal cell polarization. *Dev. Cell* 13, 609-622. 10.1016/j.devcel.2007.10.007 17981131 PMC2964935

[DEV199743C18] Gonda, M. A. and Hsu, Y.-C. (1980). Correlative scanning electron, transmission electron, and light microscopic studies of the in vitro development of mouse embryos on a plastic substrate at the implantation stage. *J. Embryol. Exp. Morphol.* 56, 23-39. 10.1242/dev.56.1.23 6893207

[DEV199743C19] Granchi, C., Bertini, S., Macchia, M. and Minutolo, F. (2010). Inhibitors of lactate dehydrogenase isoforms and their therapeutic potentials. *Curr. Med. Chem.* 17, 672-697. 10.2174/092986710790416263 20088761

[DEV199743C20] Guillomot, M., Turbe, A., Hue, I. and Renard, J.-P. (2004). Staging of ovine embryos and expression of the T-box genes Brachyury and Eomesodermin around gastrulation. *Reproduction* 127, 491-501. 10.1530/rep.1.00057 15047940

[DEV199743C21] Hashizume, K., Ushizawa, K., Patel, O. V., Kizaki, K., Imai, K., Yamada, O., Nakano, H. and Takahashi, T. (2007). Gene expression and maintenance of pregnancy in bovine: roles of trophoblastic binucleate cell-specific molecules. *Reprod. Fertil. Dev.* 19, 79-90. 10.1071/RD06118 17389137

[DEV199743C23] Hoffman, L. H. and Wooding, F. B. P. (1993). Giant and binucleate trophoblast cells of mammals. *J. Exp. Zool.* 266, 559-577. 10.1002/jez.1402660607 8371098

[DEV199743C24] Holm, P., Booth, P. J., Schmidt, M. H., Greve, T. and Callesen, H. (1999). High bovine blastocyst development in a static in vitro production system using SOFaa medium supplemented with sodium citrate and myo-inositol with or without serum-proteins. *Theriogenology* 52, 683-700. 10.1016/S0093-691X(99)00162-4 10734366

[DEV199743C25] Hsu, Y.-C. (1979). In vitro development of individually cultured whole mouse embryos from blastocyst to early somite stage. *Dev. Biol.* 68, 453-461. 10.1016/0012-1606(79)90217-3 437334

[DEV199743C26] Krisher, R. L. and Prather, R. S. (2012). A role for the Warburg effect in preimplantation embryo development: metabolic modification to support rapid cell proliferation. *Mol. Reprod. Dev.* 79, 311-320. 10.1002/mrd.22037 22431437 PMC3328638

[DEV199743C27] Lamas-Toranzo, I., Galiano-Cogolludo, B., Cornudella-Ardiaca, F., Cobos-Figueroa, J., Ousinde, O. and Bermejo-Álvarez, P. (2019). Strategies to reduce genetic mosaicism following CRISPR-mediated genome edition in bovine embryos. *Sci. Rep.* 9, 14900. 10.1038/s41598-019-51366-8 31624292 PMC6797768

[DEV199743C28] Macklon, N. S., Geraedts, J. P. and Fauser, B. C. (2002). Conception to ongoing pregnancy: the ‘black box’ of early pregnancy loss. *Hum. Reprod. Update* 8, 333-343. 10.1093/humupd/8.4.333 12206468

[DEV199743C29] Maddox-Hyttel, P., Alexopoulos, N. I., Vajta, G., Lewis, I., Rogers, P., Cann, L., Callesen, H., Tveden-Nyborg, P. and Trounson, A. (2003). Immunohistochemical and ultrastructural characterization of the initial post-hatching development of bovine embryos. *Reproduction* 125, 607-623. 10.1530/rep.0.1250607 12683931

[DEV199743C30] Moraes, J. G. N., Behura, S. K., Geary, T. W., Hansen, P. J., Neibergs, H. L. and Spencer, T. E. (2018). Uterine influences on conceptus development in fertility-classified animals. *Proc. Natl. Acad. Sci. USA* 115, E1749-E1758. 10.1073/pnas.1721191115 29432175 PMC5828633

[DEV199743C31] Negrón-Pérez, V. M., Zhang, Y. and Hansen, P. J. (2017). Single-cell gene expression of the bovine blastocyst. *Reproduction* 154, 627-644. 10.1530/REP-17-0345 28814615 PMC5630521

[DEV199743C32] Oestrup, O., Hall, V., Petkov, S. G., Wolf, X. A., Hyldig, S. and Hyttel, P. (2009). From zygote to implantation: morphological and molecular dynamics during embryo development in the pig. *Reprod. Domestic Anim.=Zuchthygiene* 44 Suppl. 3, 39-49. 10.1111/j.1439-0531.2009.01482.x 19660079

[DEV199743C33] Perez-Gomez, A., Gonzalez-Brusi, L., Bermejo-Alvarez, P. and Ramos-Ibeas, P. (2021). Lineage differentiation markers as a proxy for embryo viability in farm ungulates. *Front. Veter. Sci.* 8. 10.3389/fvets.2021.680539 PMC8239129 34212020

[DEV199743C34] Ramos-Ibeas, P., Sang, F., Zhu, Q., Tang, W. W. C., Withey, S., Klisch, D., Wood, L., Loose, M., Surani, M. A. and Alberio, R. (2019). Pluripotency and X chromosome dynamics revealed in pig pre-gastrulating embryos by single cell analysis. *Nat. Commun.* 10, 500. 10.1038/s41467-019-08387-8 30700715 PMC6353908

[DEV199743C35] Ramos-Ibeas, P., Lamas-Toranzo, I., Martínez-Moro, Á., de Frutos, C., Quiroga, A. C., Zurita, E. and Bermejo-Alvarez, P. (2020). Embryonic disc formation following post-hatching bovine embryo development in vitro. *Reproduction* 160, 579-589. 10.1530/REP-20-0243 32698149 PMC7497357

[DEV199743C36] Ribeiro, E. S., Greco, L. F., Bisinotto, R. S., Lima, F. S., Thatcher, W. W. and Santos, J. E. (2016). Biology of preimplantation conceptus at the onset of elongation in dairy cows. *Biol. Reprod.* 94, 97. 10.1095/biolreprod.115.134908 26935601

[DEV199743C37] Rodriguez, A., Allegrucci, C. and Alberio, R. (2012). Modulation of pluripotency in the porcine embryo and iPS cells. *PLoS ONE* 7, e49079. 10.1371/journal.pone.0049079 23145076 PMC3493503

[DEV199743C38] Schultz, M. L., Fawaz, M. V., Azaria, R. D., Hollon, T. C., Liu, E. A., Kunkel, T. J., Halseth, T. A., Krus, K. L., Ming, R., Morin, E. E. et al. (2019). Synthetic high-density lipoprotein nanoparticles for the treatment of Niemann-Pick diseases. *BMC Med.* 17, 200. 10.1186/s12916-019-1423-5 31711490 PMC6849328

[DEV199743C39] Seedorf, U., Leberer, E., Kirschbaum, B. J. and Pette, D. (1986). Neural control of gene expression in skeletal muscle. Effects of chronic stimulation on lactate dehydrogenase isoenzymes and citrate synthase. *Biochem. J.* 239, 115-120. 10.1042/bj2390115 2432887 PMC1147247

[DEV199743C40] Shahbazi, M. N. and Zernicka-Goetz, M. (2018). Deconstructing and reconstructing the mouse and human early embryo. *Nat. Cell Biol.* 20, 878-887. 10.1038/s41556-018-0144-x 30038253

[DEV199743C41] Shahbazi, M. N., Jedrusik, A., Vuoristo, S., Recher, G., Hupalowska, A., Bolton, V., Fogarty, N. M., Campbell, A., Devito, L. G., Ilic, D. et al. (2016). Self-organization of the human embryo in the absence of maternal tissues. *Nat. Cell Biol.* 18, 700-708. 10.1038/ncb3347 27144686 PMC5049689

[DEV199743C42] Shahbazi, M. N., Wang, T., Tao, X., Weatherbee, B. A. T., Sun, L., Zhan, Y., Keller, L., Smith, G. D., Pellicer, A., Scott, R. T. et al. (2020). Developmental potential of aneuploid human embryos cultured beyond implantation. *Nat. Commun.* 11, 3987. 10.1038/s41467-020-17764-7 32778678 PMC7418029

[DEV199743C43] Simintiras, C. A., Sánchez, J. M., McDonald, M. and Lonergan, P. (2019). Progesterone alters the bovine uterine fluid lipidome during the period of elongation. *Reproduction* 157, 399-411. 10.1530/REP-18-0615 30763281

[DEV199743C44] Solnica-Krezel, L. and Sepich, D. S. (2012). Gastrulation: making and shaping germ layers. *Annu. Rev. Cell Dev. Biol.* 28, 687-717. 10.1146/annurev-cellbio-092910-154043 22804578

[DEV199743C45] Spanos, S., Becker, D. L., Winston, R. M. and Hardy, K. (2000). Anti-apoptotic action of insulin-like growth factor-I during human preimplantation embryo development. *Biol. Reprod.* 63, 1413-1420. 10.1095/biolreprod63.5.1413 11058546

[DEV199743C46] Spindle, A. (1980). An improved culture-medium for mouse blastocysts. *In Vitro-J. Tissue Cult. Assoc.* 16, 669-674. 10.1007/BF02619196 7419236

[DEV199743C48] Tian, G.-P., Chen, W.-J., He, P.-P., Tang, S.-L., Zhao, G.-J., Lv, Y.-C., Ouyang, X.-P., Yin, K., Wang, P.-P., Cheng, H. et al. (2012). MicroRNA-467b targets LPL gene in RAW 264.7 macrophages and attenuates lipid accumulation and proinflammatory cytokine secretion. *Biochimie* 94, 2749-2755. 10.1016/j.biochi.2012.08.018 22963823

[DEV199743C49] Vajta, G., Alexopoulos, N. I. and Callesen, H. (2004). Rapid growth and elongation of bovine blastocysts in vitro in a three-dimensional gel system. *Theriogenology* 62, 1253-1263. 10.1016/j.theriogenology.2004.01.007 15325552

[DEV199743C50] van Leeuwen, J., Berg, D. K. and Pfeffer, P. L. (2015). Morphological and gene expression changes in cattle embryos from hatched blastocyst to early gastrulation stages after transfer of in vitro produced embryos. *PLoS ONE* 10, e0129787. 10.1371/journal.pone.0129787 26076128 PMC4468082

[DEV199743C51] van Leeuwen, J., Rawson, P., Berg, D. K., Wells, D. N. and Pfeffer, P. L. (2020). On the enigmatic disappearance of Rauber's layer. *Proc. Natl. Acad. Sci. USA* 117, 16409-16417. 10.1073/pnas.2002008117 32601185 PMC7368265

[DEV199743C52] Wang, Y.-P., Zhou, L.-S., Zhao, Y.-Z., Wang, S.-W., Chen, L.-L., Liu, L.-X., Ling, Z.-Q., Hu, F.-J., Sun, Y.-P., Zhang, J.-Y. et al. (2014). Regulation of G6PD acetylation by SIRT2 and KAT9 modulates NADPH homeostasis and cell survival during oxidative stress. *EMBO J.* 33, 1304-1320. 10.1002/embj.201387224 24769394 PMC4194121

[DEV199743C53] Wiley, L. M. and Pedersen, R. A. (1977). Morphology of mouse egg cylinder development in vitro: light and electron-microscopic study. *J. Exp. Zool.* 200, 389-402. 10.1002/jez.1402000309 327020

[DEV199743C54] Wimsatt, W. A. (1951). Observations on the morphogenesis, cytochemistry, and significance of the binocleate giant cells of the placenta of ruminants. *Am. J. Anat.* 89, 233-281. 10.1002/aja.1000890204 14894441

[DEV199743C55] Wooding, F. B. (1982). The role of the binucleate cell in ruminant placental structure. *J. Reprod. Fertil. Suppl.* 31, 31-39. 6762432

[DEV199743C56] Xiang, L., Yin, Y., Zheng, Y., Ma, Y., Li, Y., Zhao, Z., Guo, J., Ai, Z., Niu, Y., Duan, K. et al. (2020). A developmental landscape of 3D-cultured human pre-gastrulation embryos. *Nature* 577, 537-542. 10.1038/s41586-019-1875-y 31830756

[DEV199743C57] Zhou, F., Wang, R., Yuan, P., Ren, Y., Mao, Y., Li, R., Lian, Y., Li, J., Wen, L., Yan, L. et al. (2019). Reconstituting the transcriptome and DNA methylome landscapes of human implantation. *Nature* 572, 660-664. 10.1038/s41586-019-1500-0 31435013

