# Supplementary material for: In vitro culture of ovine embryos up to early gastrulating stages
Source: Development. 2022 Mar 24;149(6):dev199743. doi: 10.1242/dev.199743 (PMC8977095; doi:10.1242/dev.199743)
Supplement: Supplementary information [file develop-149-199743-s1.pdf]

Supplementary materials and methods

In vivo recovery of ovine embryos

All experimental procedures were approved by INIA Animal Care Committee and Madrid Region Authorities (PROEX 040/17) in agreement with European legislation. *In vivo* embryos were obtained from 3 years old Merino ewes superovulated following the protocol described in **Fig. S8**, which involved flugestone acetate (Sincropart® sponges, CEVA), PGF2α analog (Cloprostenol, Estrumate®, MSD) and follicle-stimulating hormone (Folltropin-V, Vetoquinol). Ewes were mated (day 0 PM and day 1 AM) and slaughtered at days 11, 12.5 and 14 post-coitus. Embryos were recovered by uterine flushing with warmed recovery medium (Euroflush, IVM Technologies).

In vitro production of ovine blastocysts

Immature cumulus oocyte complexes (COCs) were obtained by aspirating follicles (2 to 8 mm diameter) from ovine ovaries collected at a local slaughterhouse with a 23G needle connected to an aspiration pump (VMAR 5100, Cook) adjusted to -25 mmHg. COCs were collected in a 50 ml Falcon® tube (Becton Dickinson) with TCM-199 supplemented with 40 µg/ml gentamicin sulphate, 10 mM HEPES, 10 IU/ml heparin to prevent clotting and 4 mM hypoxanthine (Sigma) to prevent meiotic resumption. COCs with a compact cumulus and homogeneous cytoplasm were selected and washed in with TCM-199 supplemented with 40 µg/ml gentamicin sulphate and 10 mM HEPES, and then matured for 24 h in TCM-199 supplemented with 40 µg/ml gentamicin sulphate, 10 % (v/v) fetal bovine serum (FBS), 16 IU/ml FSH, 7.8 IU/ml LH, 100 µM cysteamine, 1 µg/ml 17 β-oestradiol and 10 ng/ml epidermal growth factor (EGF) at 39 °C under an atmosphere of 5 % CO_2 in air with maximum humidity. For *in vitro* fertilization (IVF), matured COCs were partially denuded by gentle pipetting and inseminated with frozen-thawed Bovi-Pure® (Nidacon) separated ram sperm at a final concentration of 10^6 spermatozoa/ml. Gametes were co-incubated in 50 µl droplets of synthetic oviductal fluid (SOF) medium supplemented with 20% (v/v) heat-inactivated oestrous sheep serum covered by mineral oil and containing 25 COCs (1) at 38.5 °C in an atmosphere of 5 % CO_2 and maximum humidity. Semen from the same ram was used for all the experiments to avoid a possible confounding ram effect on developmental rates. At approximately 20 h post-insemination (hpi), presumptive zygotes were denuded and cultured in groups of 25 in 50 µl droplets under mineral oil. Culture took place in SOF supplemented with 0.3% bovine serum albumin (BSA) and 40 µg/ml gentamicin sulphate at 38.5 °C under an atmosphere of 5 % CO_2, 5 % O_2, and 90 % N_2 with maximum humidity. Cleavage was assessed at 48 h, and at day 4 after IVF, embryos were transferred to SOF supplemented with 10 % (v/v) FBS.

Development • Supplementary information

Post-hatching development system

Blastocysts developed at days 6 and 7 after IVF were transferred to agarose-coated four-well dishes in groups of 10-15. Agarose-coated dishes were prepared 3 days before use. Gel was prepared by solving 2.4 % ultrapure low melting point agarose (Thermo Fisher Scientific) in PBS. The solution was autoclaved and poured into the wells once cold down to 45 ºC. Dishes were placed on ice bags for rapid solidification of the gel, and then culture medium was poured on the gel surface. Dishes were kept at 38.5 ºC and 5 % CO_2 in air, and medium was replaced daily until dishes were used for embryo culture (2). In a first experiment, blastocysts were randomly allocated to three different culture media: 1) SOF supplemented with 10% (v/v) FBS; 2) an *in vitro* culture medium supporting post-blastocyst development in human embryos (hIVC) (3), composed by IVC1 medium [DMEM/F12 medium supplemented with 2 mM L-glutamine, 1x Insulin-Transferrin-Selenium-Ethanolamine (ITS-X, Thermo Fisher Scientific), 8 nM β-estradiol, 200 ng/ml progesterone, 25 μM N-acetylcysteine and 20% FBS] during the first 2 days of embryo culture, gradually replaced by IVC2 medium [DMEM/F12 medium supplemented with 2 mM L-glutamine, 1x Insulin-Transferrin-Selenium-Ethanolamine (ITS-X, Thermo Fisher Scientific), 8 nM β-estradiol, 200 ng/ml progesterone, 25 μM N-acetylcysteine and 30% Knockout Serum Replacement (KSR, Thermo Fisher Scientific)] from the third day of culture; and 3) N2B27 medium [1:1 Neurobasal and DMEM/F12 medium supplemented with penicillin/streptomycin, 2 mM L-glutamine, N2 and B27 supplements (Thermo Fisher Scientific)].

In subsequent experiments, blastocysts were randomly cultured in hIVC alone or supplemented with 10 μM Rho-associated protein kinase (ROCK) inhibitor (Y-27632, Stem Cell Technologies); or in N2B27 alone or supplemented with 10 μM ROCK inhibitor, 20 ng/ml activin A (Stem Cell Technologies), 100 ng/ml Insulin Growth Factor 1 (IGF1, Thermo Fisher Scientific) or 20 ng/ml basic Fibroblast Growth Factor (bFGF, Thermo Fisher Scientific), or a combination of 10 μM ROCK inhibitor and 20 ng/ml activin A.

All post-hatching development conditions were tested at 38.5 ºC in a water saturated atmosphere of 5 % CO_2, 5 % O_2, and 90 % N_2 and half of the culture medium was replaced every other day. At the end of the culture at D14, pictures from the embryos were taken on a stereo microscope (Zeiss Stemi 305) and embryo area was measured using Fiji software (4). Embryo survival was analyzed following similar criteria than under conventional culture up to expanded blastocysts: alive embryos were able to maintain the blastocoel, whereas dead embryos collapsed (Fig. S10A). Surviving embryos were collected for further analyses.

Immunofluorescence and lineage development analysis

Embryos were fixed in 4 % paraformaldehyde (PFA) for 15 minutes at room temperature (RT), washed in PBS - 1 % BSA, permeabilized in 1 % Triton X-100 in PBS for 15 min at RT and blocked in 10 % Donkey Serum-0.02 % Tween 20 in PBS for 1 h at RT. Then, embryos were incubated overnight at 4 ºC with primary antibodies to detect epiblast (SOX2), hypoblast (SOX17), mesoderm

Development • Supplementary information

(BRACHYURY), trophectoderm (GATA3), the principal kinase of the apical Par polarity complex (aPKC) or basal membrane (LAMININ). After 4 washes in PBS-1% BSA, embryos were incubated in the appropriate secondary Alexa-conjugated antibodies or in Alexa 488 Phalloidin to detect F-actin (**Table S3**) and counterstained with DAPI for 1 h at RT, followed by 4 washes in PBS-1 % BSA. Finally, embryos were mounted and imaged at a structured illumination equipment composed by a Zeiss Axio Observer microscope coupled to ApoTome.2 or at a fluorescence stereomicroscope (Zeiss V20). For tridimensional images, embryos were placed on PBS - 1 % BSA microdrops made by drawing circles with a PAP pen (Kisker Biotech GmbH) on a coverslide as previously described (5). Microdrops were covered by an incubation chamber (Sigma Z37,9467) to prevent embryo crushing. Cells were counted using the ZEN software (Zeiss). Following immunofluorescence analysis, lineage development was analyzed. Epiblast survival was identified by the presence of SOX2+ cells in the embryo (Fig. S10b, d-f), whereas ED formation was identified by the presence of a compact structure of at least 30 SOX2+ cells (Fig. S10d-f). Hypoblast migration was considered complete when all the inner surface of the trophectoderm was covered by SOX17+ cells (Fig. S10b-c).

Apoptotic cells detection

The TdT-mediated dUTP-biotin Nick end-labeling (TUNEL) assay was employed for apoptotic cell detection using the In Situ Cell Death Detection Kit, TMR Red (Roche) according to the manufacturer’s instructions with minor modifications. Briefly, after fixation, embryos were permeabilized in 0.5% Triton X-100 and 0.1% Sodium Citrate in PBS for 20 min at RT. Then, embryos were incubated in 30 µl drops of TUNEL reaction mixture for 1 h at 37 ºC in a humidified chamber and then washed in PBS - 1 % BSA. Finally, embryos were mounted in Fluoroshield with DAPI in slides with 8 mm diameter rings and round coverslips (Thermo Fisher). Embryos were imaged at the structured illumination equipment previously described. Z-stack images were taken to detect all apoptotic cells along the Z axis. Cells were counted using the Fiji software (4) and apoptotic rate was determined by calculating the ratio of the total number of TUNEL positive cells/number total cells in the embryo.

RNA isolation, cDNA synthesis and qPCR

Poly (A) RNA was extracted from 4 individual whole D14 embryos of each group and 4 pools of 10 D7 blastocysts using the Dynabeads mRNA Purification Kit (Life Technologies, Oslo, Norway) following the manufacturer’s instructions with minor modifications (6). Briefly, 50 µl of lysis buffer were added to the sample and incubated at RT for 10 min with gently shaking. Then, 10 µl of beads were added and samples were incubated at RT for 5 min with gentle shaking, allowing beads/mRNA complexes formation. Finally, beads/mRNA complexes were washed twice in washing buffer A and twice in washing buffer B, and resuspended in 10 mM Tris-HCl pH 7.5. The

Development • Supplementary information

amount of mRNA/sample was roughly similar, being around 4 ng. Immediately after extraction, samples were treated with DNAse (Promega, Madison, WI, USA) at 37 ºC for 5 min followed by enzyme denaturalization at 90 ºC for 5 minutes, and then the reverse transcription reaction was carried out with qScript cDNA Supermix (Quantabiosciences, Gaithersburg, MS, USA) in a total volume of 20 µl. Tubes were first incubated at 25°C for 5 min and then at 42°C for 60 min to allow the reverse transcription of RNA, followed by 85°C for 5 min to denature the reverse transcriptase. mRNA transcripts were quantified by real-time quantitative PCR (qPCR). Two replicate PCR experiments were conducted for all genes of interest and qPCR efficiency was tested beforehand, all primers used showing efficiencies above 0.9. PCR was performed by adding a 2-µl aliquot of each sample to the PCR mix (GoTaq qPCR Master Mix, Promega, Madison, WI, USA) containing the specific primers. Primer sequences are provided in **Table S4**. The comparative cycle threshold (CT) method was used to quantify expression levels. Fluorescence was acquired in each cycle to determine the threshold cycle. According to the comparative CT method, the CT value was determined by subtracting the endogenous control *H2AFZ* CT value (7) for each sample from the CT value of each gene in the sample. CT was calculated using the highest sample CT value (i.e., the sample with the lowest target expression) as an arbitrary constant to be subtracted from all other CT sample values. Fold changes in the relative gene expression of the target were determined using the formula $2^{-\Delta\Delta CT}$ (8).

RNA sequencing

Total RNA was extracted from 3 D14 *in vitro*, 3 E11 and 3 E12.5 *in vivo* embryos using MagMAX™ *mir*Vana™ Total RNA Isolation kit according to the manufacturer´s protocol. cDNA was synthetized with SMART-Seq™ v4 Ultra™ Low Input RNA Kit (Clontech) and amplified ds-cDNA was purified with AMPure XP beads (Beckman Coulter) and quantified with Qubit (Life Technologies). Libraries were prepared using Covaris shearing system and a size selection of 200 bp was performed. Library concentration was first quantified using Qubit and then diluted to 2 ng/µl before checking insert size on an Agilent 2100 and quantifying by qPCR. Libraries were pooled and sequenced on a HiSeq 2500 Sequencing System (Illumina).

The number of raw reads (150-bp paired-end reads) ranged from 40 to 63 million per sample. Paired-end read fastq files were quality checked with FastQC (9) and preprocessed with fastp (10). Resulting files were pseudoaligned and quantified using kallisto (11) against the reference transcriptome of *Ovis aries* Rambouillet breed v1.0 (Ensembl release 104). Differential gene expression analysis was performed with R (v4.1.2), with the package DESeq2 (12) and collapsing transcript expression data to the gene level with tximport (13). A gene was considered as differentially expressed (DEG) between two experimental groups if its p-adj < 0.01 and its shrunken fold change > 2. To perform gene annotation of the DEGs, the mart database “oarambouillet_gene_ensembl” from the R package biomaRt (14) was used. An ortholog search

Development • Supplementary information

was done to assign gene names to those ensembl IDs without an associated gene name. This step was performed with the eggNOG mapper (15, 16) deploying blastx-like searches against a DIAMOND (17) database comprised of curated mammalian proteins. RNA-seq datasets generated during this study are available under GEO accession number: GSE189360.

Data and statistical analysis

Data analysis was blinded and manually performed by two different researchers with homogeneous criteria after testing for differences. Representative examples from each category (embryo survival, complete hypoblast migration, epiblast survival and embryonic disc formation) are provided in **Fig. S9**. Epiblast survival was scored as the presence of SOX2+ cells at D14. SOX2+ cell number, as well as TUNEL+ and total cell number, were counted manually using the multi-point counter plugin in ZEN 3.2 (Carl Zeiss, Germany).

Data were analysed using the GraphPad Prism (GraphPad Software, San Diego, CA, USA) and Sigmastat (Systat Software, San Jose, CA, USA) packages and a value of $P < 0.05$ was considered significant. Chi-square test was used to analyse the differences in embryo survival, complete hypoblast migration, epiblast survival and embryonic disc formation between groups. Differences in area and apoptotic cells rate between groups were analysed by Student´s t-test when data distribution was normal. When normality test failed, statistical differences were analysed by Mann-Whitney Rank Sum Test. Differences in mRNA expression, embryo length, ED area and SOX2-positive cell number were analysed by One-way ANOVA. When normality test failed, statistical differences were analysed by non-parametric One-way ANOVA (Kruskal-Wallis test). Additionally, SOX2-positive cell number was analysed by non-parametric bootstrapping with the R package nptest (version 1.0-3) with 1000 replicates per statistical test, and dependence on outliers was discarded (Fig. 2B: P-value for N2B27 vs. N2B27+R = 0.01; P-value for N2B27 vs. N2B27+A = 0.003. Fig. 2E: P-value for N2B27 vs. N2B27+A+R = 0.001; Fig. 3D: P-value for D14 vs. E11 = 0.0003; P-value for D14 vs. E12.5 = 0.045; P-value for D14 vs. E14 = 0.0001).

Development • Supplementary information

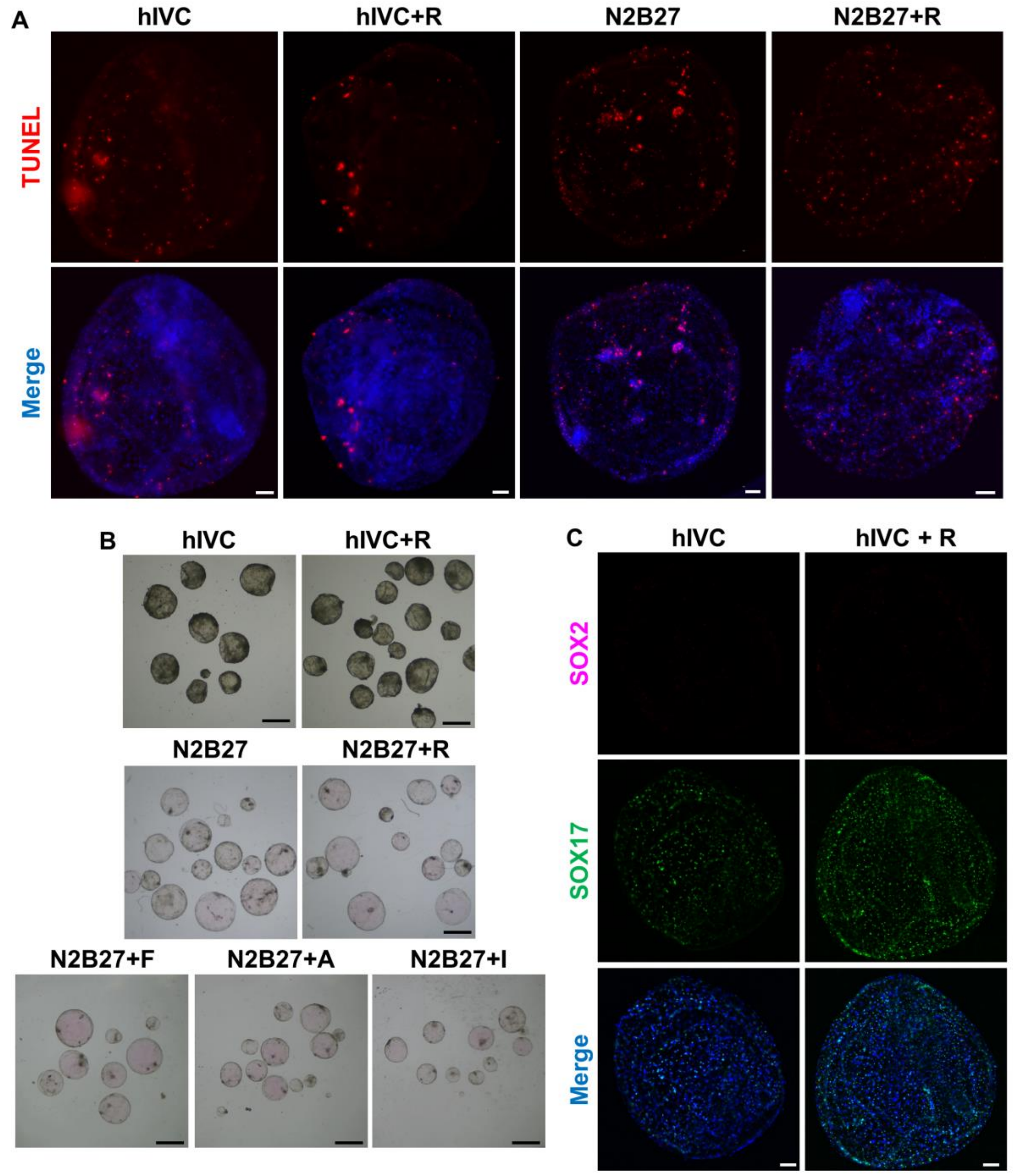

Fig. S1. Establishing a post-hatching culture system for sheep embryos. (a) Representative fluorescence images of D14 embryos developed in hIVC alone or supplemented with ROCK inhibitor, N2B27 alone or supplemented with ROCK inhibitor, stained for apoptotic cells with TUNEL; nuclei were counterstained with DAPI (merge). (b) Representative brightfield stereomicroscopic images of D14 embryos cultured in hIVC, hIVC supplemented with 10 µM Y27632 (ROCK inhibitor, hIVC+R), N2B27 alone or supplemented with 10 µM Y27632 (ROCK inhibitor, N2B27+R), 20 ng/ml bFGF (N2B27+F), 20 ng/ml activin A (N2B27+A) or 100 ng/ml IGF1 (N2B27+I). (c) Representative fluorescence images of D14 embryos developed in hIVC alone or supplemented with ROCK inhibitor stained for SOX2 (epiblast) and SOX17 (hypoblast); nuclei were counterstained with DAPI (merge). Scale bars = 1 mm for b; 100 µm for a and c.

Development • Supplementary information

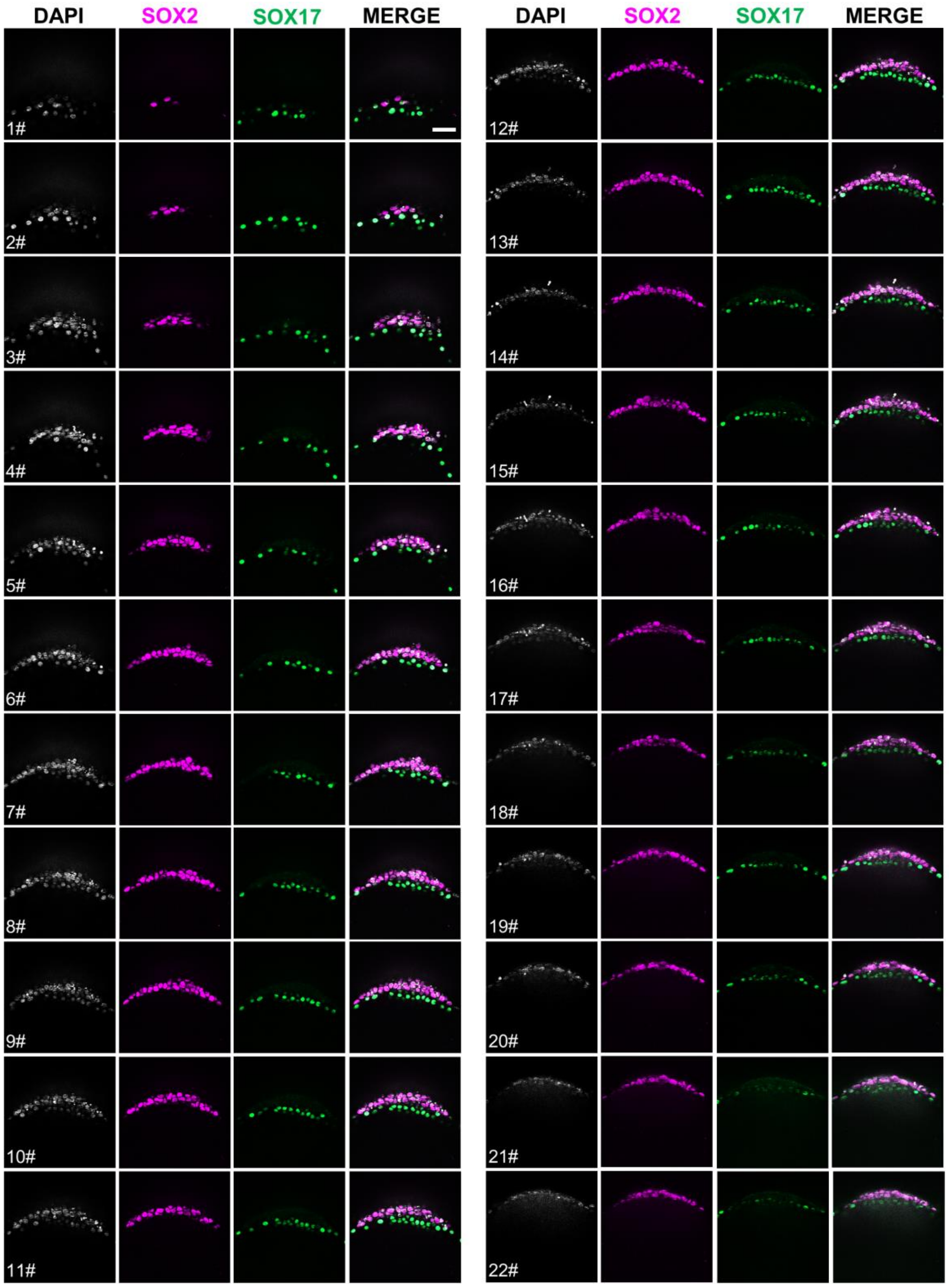

Fig. S2. Representative Z-series of an ED without Rauber´s layer from a D14 *in vitro* embryo cultured in N2B27 + A + R. This figure is related to Figure 2d. Series of confocal z-sections of the D14 embryo stained for SOX2 (magenta) and SOX17 (green); nuclei were counterstained with DAPI (white). Note the absence of trophoblast cells (white) over the epiblast. The thickness of every section was 5 µm. Numbers on the left indicate the number of sections. Similar phenotype was observed in 22 out of 36 embryos. Scale bar = 50 µm.

Development • Supplementary information

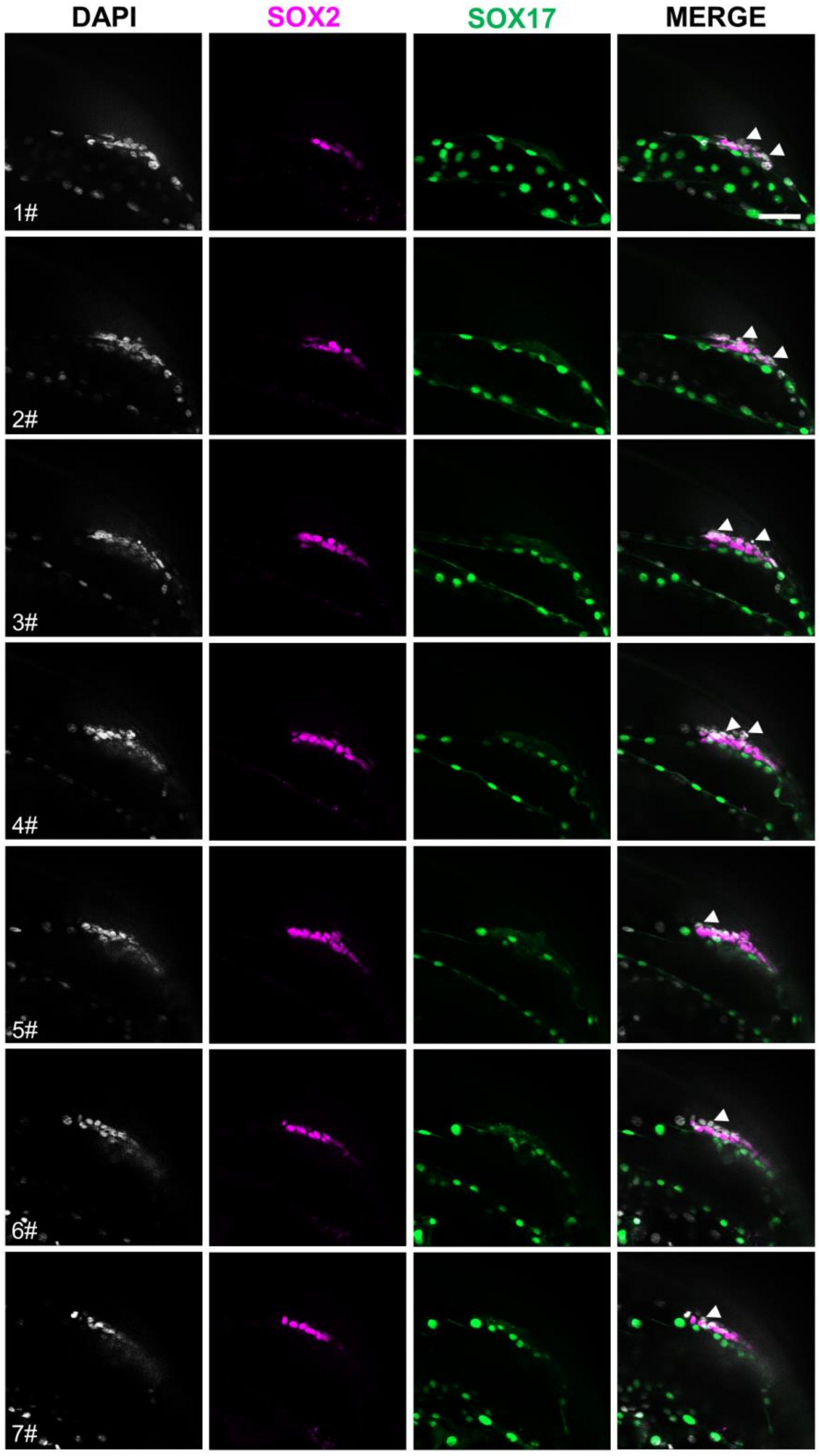

Fig. S3. Representative Z-series of an ED with Rauber´s layer from a D14 *in vitro* embryo cultured in N2B27. This figure is related to Figure 2d. Series of confocal z-sections of an embryo stained for SOX2 (magenta) and SOX17 (green); nuclei were counterstained with DAPI (white). Arrowheads indicate trophoblast cells (white) over the epiblast. The thickness of every section was 5 µm. Numbers on the left indicate the number of sections. Similar phenotype was observed in 6 out of 6 embryos. Scale bar = 50 µm.

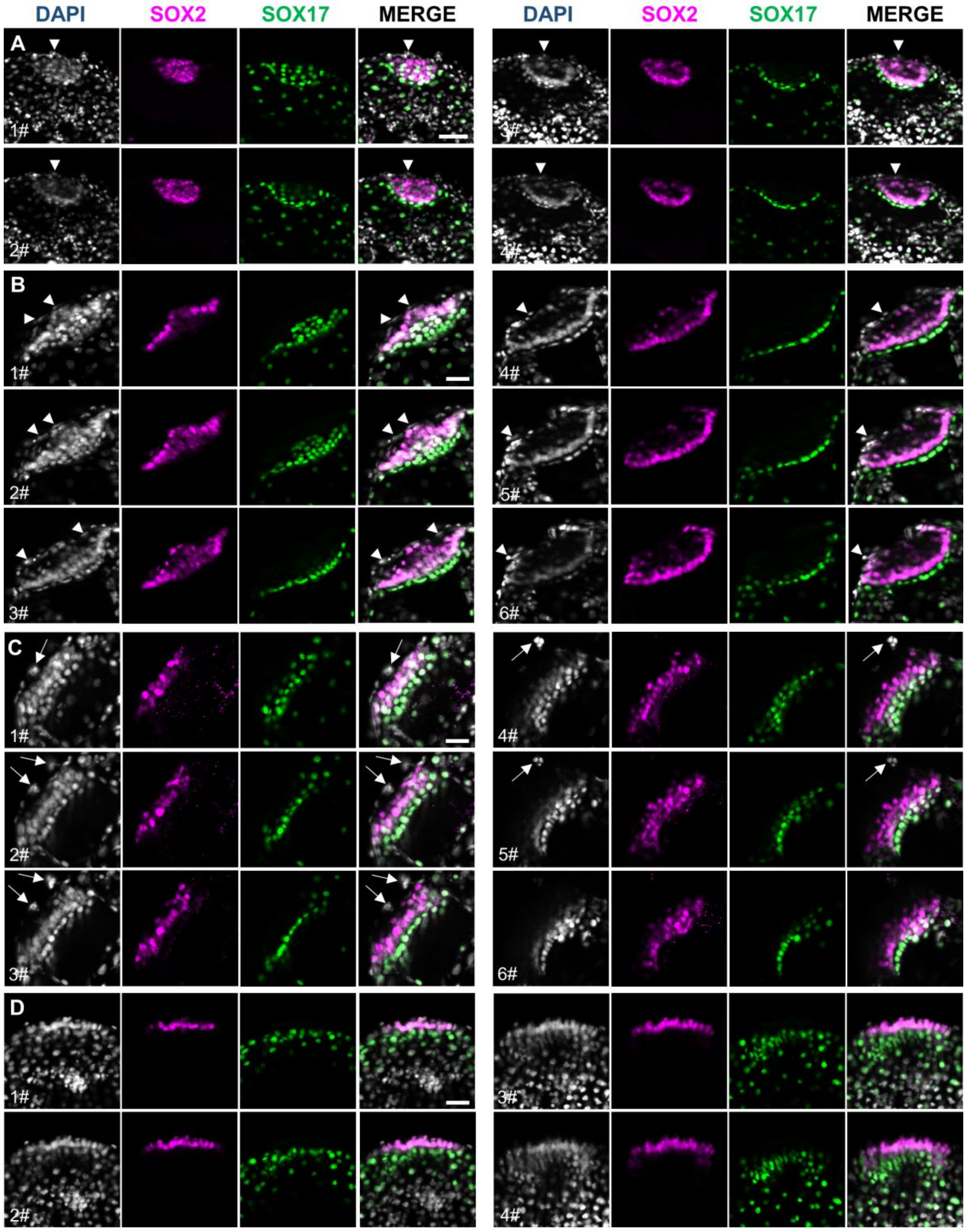

Fig. S4. Representative Z-series of EDs from E11 *in vivo*-derived embryos. Series of confocal z-sections of EDs (a,b) with Rauber´s layer, (c) with the Rauber´s layer being removed and (d) without Rauber´s layer, stained for SOX2 (magenta) and SOX17 (green); nuclei were counterstained with DAPI (white). Note epiblast cavitation in a (sections #4 and #5) and b (sections #4 to #6). Arrowheads indicate trophoblast cells (white) over the epiblast. The thickness of every section was 5 µm. Numbers on the left indicate the number of sections. Scale bar = 50 µm.

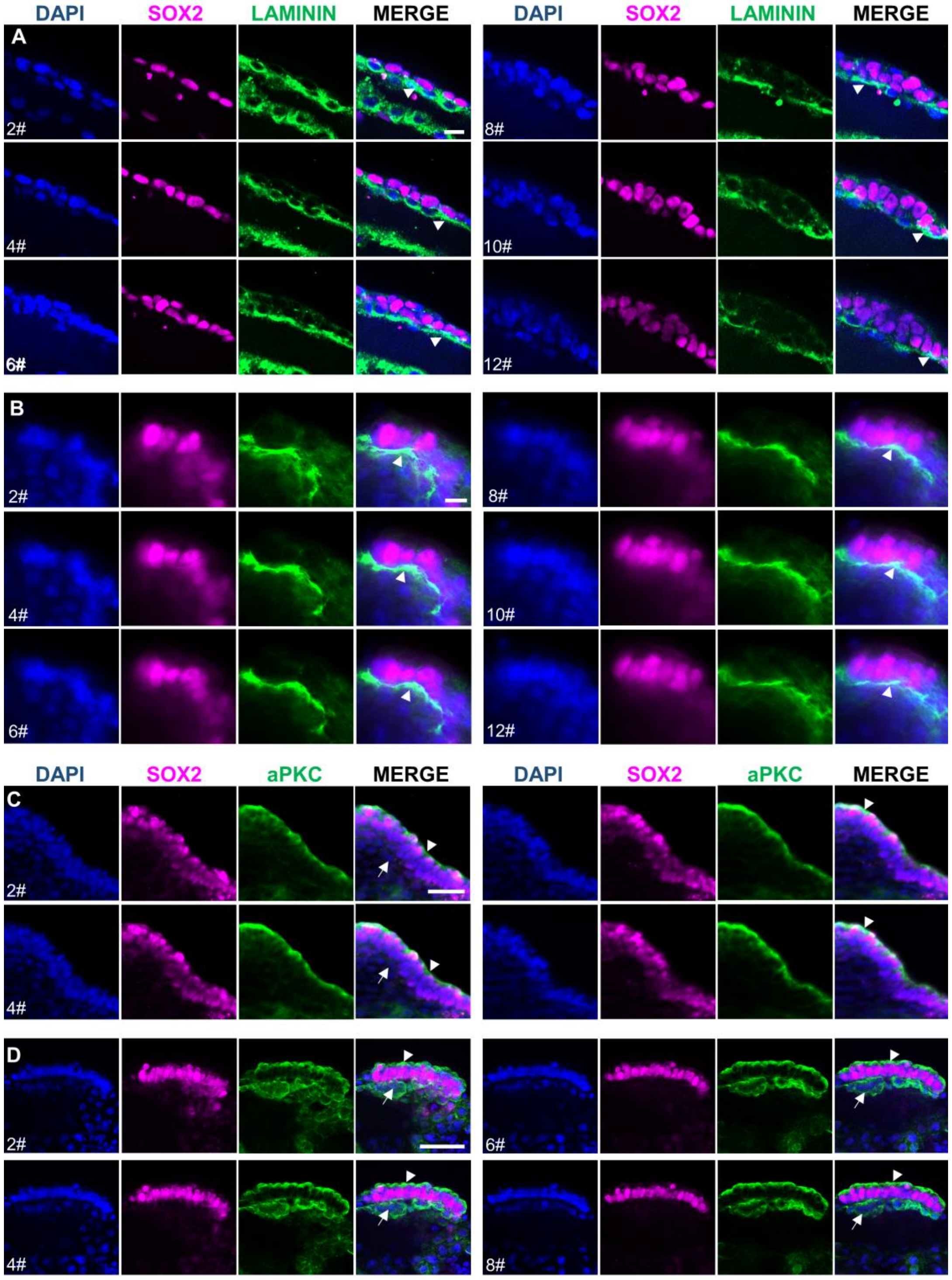

Fig. S5. Representative Z-series of EDs from (a, c) D14 *in vitro* and (b, d) E11 *in vivo*-derived embryos. This figure is related to Figures 4a and b. Series of confocal z-sections of an EDs stained for (a, b) SOX2 (magenta) and LAMININ (green); (c, d) SOX2 (magenta) and aPKC (green); nuclei were counterstained with DAPI (merge). Arrowheads indicate (a, b) laminin accumulation in the basal side of SOX2+ epiblast cells; (c, d) apical localization of aPKC. Arrows point to hypoblast cells. The thickness of every section was 5 µm. Numbers on the left indicate the number of sections. Similar phenotype was observed in (a) 7 out of 9 embryos; (b) 2 out of 2 embryos; (c) 4 out of 4 embryos; (d) 3 out of 3 embryos. Scale bars = 10 µm for a and b, 50 µm for c and d.

Development • Supplementary information

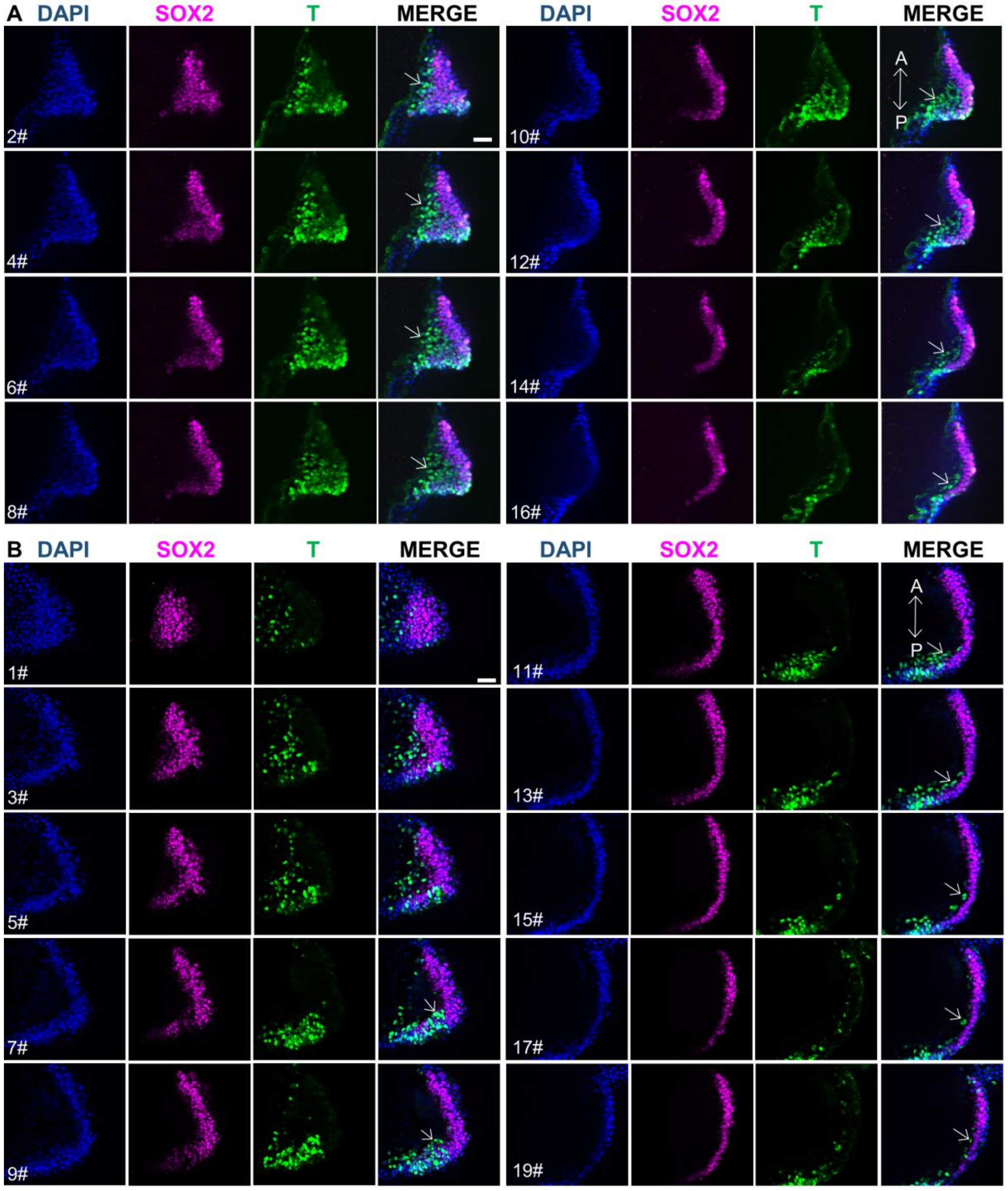

Fig. S6. Representative Z-series of (a) a D14 *in vitro* embryo and (b) a E12.5 *in vivo* embryo showing mesoderm differentiation. This figure is related to Figure 4c. Series of confocal z-sections of an embryo stained for SOX2 (magenta) and BRACHYURY (T, green) showing mesoderm differentiation. The thickness of every section was 5 µm. Numbers on the left indicate the number of sections. White arrows indicate migrating T^{+} cells. Double arrow indicates anterior-posterior (A-P) axis. Similar phenotype was observed in (a) 8 out of 18 *in vitro* embryos; (b) 6 out of 6 *in vivo* embryos. Scale bars = 50 µm.

Development • Supplementary information

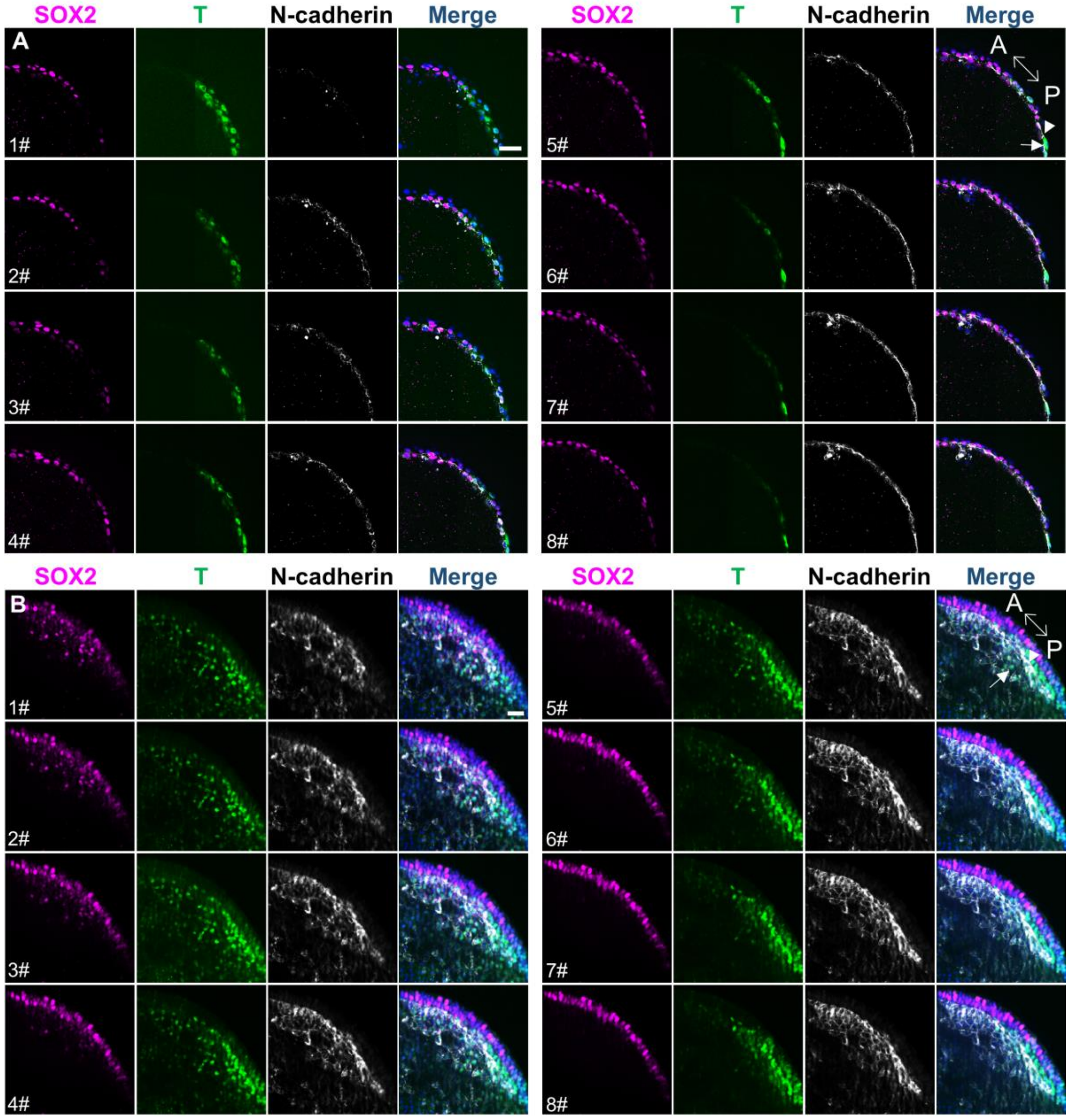

Fig. S7. Representative Z-series of (a) a D14 *in vitro* embryo and (b) a E12.5 *in vivo* embryo showing gastrulation. This figure is related to Figure 4d. Series of confocal z-sections of an embryo stained for SOX2 (magenta), BRACHYURY (T, green) and N-cadherin (white) showing mesoderm cells (T+) migrating from the ED and expression of the EMT marker N-cadherin. The thickness of every section was 5 μm. Numbers on the left indicate the number of sections. White arrows indicate migrating T^+ cells. Arrowhead indicates N-cadherin+ basement membrane. Double arrow indicates anterior-posterior (A-P) axis. Similar phenotype was observed in (a) 4 out of 9 *in vitro* embryos; (b) 5 out of 5 *in vivo* embryos. Scale bars = 50 μm.

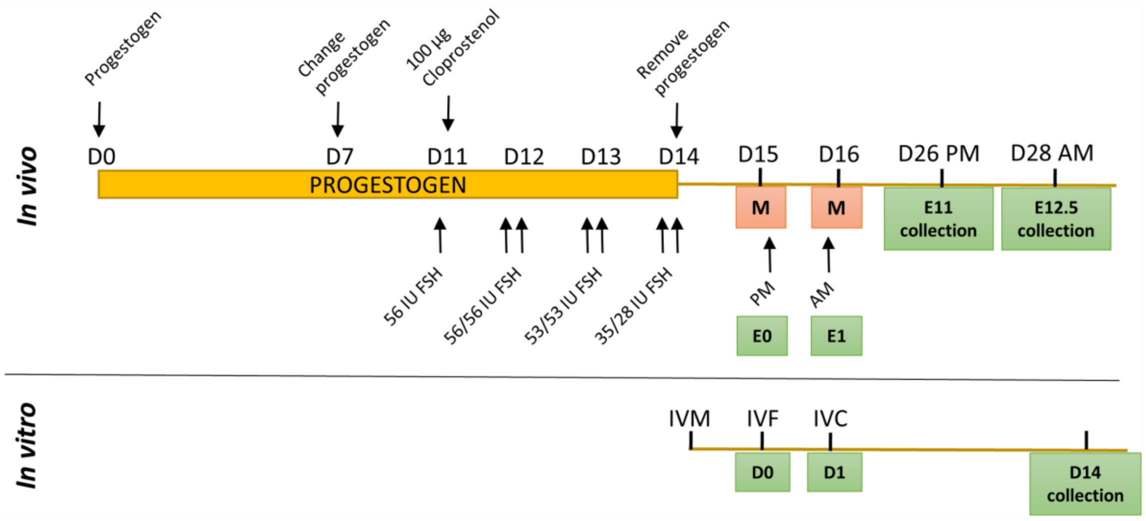

Fig. S8. Timeline for *in vivo* and *in vitro* embryo collection. Superovulation protocol employed to obtain *in vivo* derived embryos in sheep. D: days after the beginning of IVF (for *in vitro* embryos), E: embryonic day, days after mating (for *in vivo* embryos), FSH: Follicle stimulating hormone, IVM: *in vitro* maturation, IU: international units, IVC: *in vitro* culture, IVF: *in vitro* fertilization, M: mating.

Development • Supplementary information

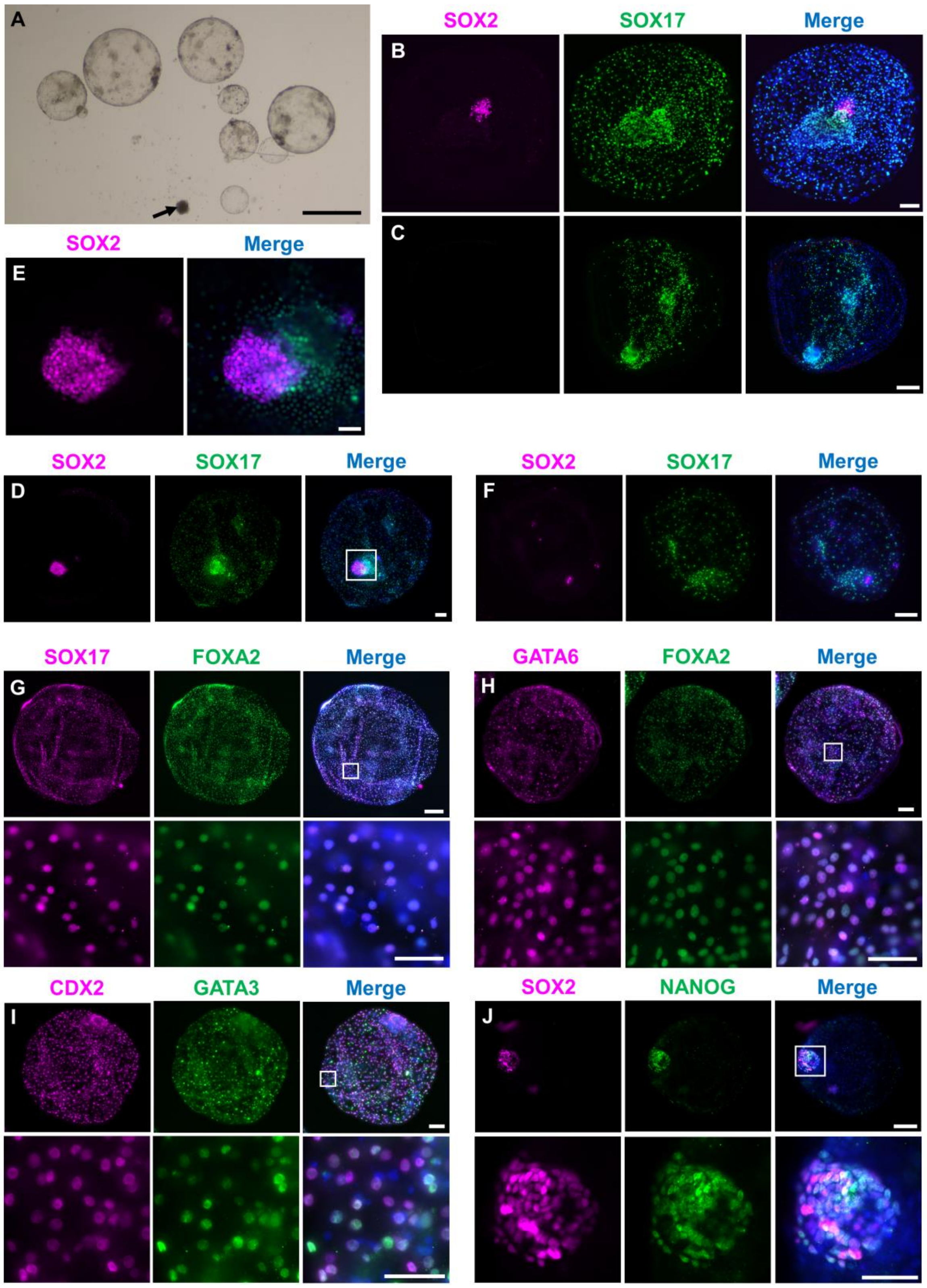

Fig S9. Representative examples of the embryonic parameters analyzed and validation of the antibodies used to analyze lineages development. (a) Representative brightfield stereomicroscopic image of D14 embryos. All embryos are alive except for the one pointed with an arrow. Complete (b) and incomplete (c) hypoblast migration along the inner embryo surface

Development • Supplementary information

analyzed by SOX17 staining. Epiblast survival denoted by the presence of SOX2+ cells in (b) but not in (c). Embryo in (d) shows ED formation (a compact structure of at least 30 SOX2+ cells); magnification in (e). Embryo in (f) shows epiblast survival but no ED formation. (g) Hypoblast cells from a D14 embryo stained for SOX17 and FOXA2. (h) Hypoblast cells from a D14 embryo stained for GATA6 and FOXA2. (i) Trophoblast cells from a D14 embryo stained for CDX2 and GATA3. (j) Epiblast cells from a D14 embryo stained for SOX2 and NANOG. Magnifications are shown for each embryo in g-j. Scale bars = 1 mm for a; 200 µm for c, g, h, i and j; 100 µm for b, d and f; 50 µm for e and magnifications in g-j.

Development • Supplementary information

Table S1. Survival, area and development of hypoblast and epiblast lineages of surviving embryos at D14 after culture in hIVC or hIVC + ROCK inhibitor.

	Survival (%)	Area (µm^2)	Complete hypoblast migration (%)	Epiblast survival (%)
hIVC	30/33 (90.9 %)	898 ± 92.81	22/24 (91.7 %)	0/24 (0%)
hIVC + R	36/40 (90 %)	799.4 ± 79.10	17/22 (77.3 %)	0/22 (0%)

Mean ± s.e.m. for area. No significant differences were found between both groups (survival, complete hypoblast migration and epiblast survival: Chi-square test; area: One-way ANOVA, Dunn´s test). R: ROCK inhibitor.

Table S2. Differentially expressed genes.

Click here to download Table S2

Development • Supplementary information

Table S3. Details of antibodies used immunostaining.

	Antigen	Host species	Company	Cat No.	RRID	Dilution
Primary	SOX2	Rat	Invitrogen	14-9811-80	AB_11219471	1:100
	SOX17	Goat	R&D	AF1924	AB_355060	1:100
	FOXA2	Rabbit	Cell Signalling	8186S	AB_10891055	1:100
	GATA3	Rabbit	Abcam	ab199428	AB_2819013	1:100
	aPKC	Mouse	Santa Cruz	sc-17781	AB_628148	1:100
	LAMININ	Rabbit	Sigma	L9393	AB_477163	1:100
	HESX1	Rabbit	Abcam	Ab246949	-	1:100
	N-CADHERIN	Rabbit	Abcam	Ab18203	AB_444317	1:100
	EOMES	Mouse	R&D	MAB6166	AB_10919889	1:100
	BRACHYURY (T)	Goat	R&D	AF2085	AB_2200235	1:100
Secondary	Anti-Rat IgG 594	Donkey	Life Technologies	A-21209	AB_2535795	1:300
	Anti-Goat IgG 488	Donkey	Life Technologies	A-32814	AB_2762838	1:300
	Anti-Rabbit IgG 488	Donkey	Life Technologies	A-32790	AB_2762833	1:300
	Anti-Rabbit IgG 594	Donkey	Life Technologies	A-32754	AB_2762827	1:300
	Anti-Mouse IgG 488	Donkey	Life Technologies	A-32766	AB_2762823	1:300
	Phalloidin 488	-	Invitrogen	A-12379	-	1:400

Development • Supplementary information

Table S4. Details of primers used for qPCR.

Gene	Primer sequence (5’→3’)	Fragment size (bp)	GeneBank accession
H2AZ1	F: AGGACGACTAGCCATGGACGTGTG<br>R: CCACCACCAGCAATTGTAGCCTTG	209	NM_001009270.1
TP-1	F: CCTACACCCGCCTGTGTTCA<br>R: AGGACTCATGCCCCTACAGC	141	NM_001123399.1
LDHA	F: TTCTTAAGGAAGAACATGTC<br>R: TTCACGTTACGCTGGACCAA	310	XM_027959818.1
GAPDH	F: ACCCAGAAGACTGTGGATGG<br>R: ACGCCTGCTTCACCACCTTC	247	NM_001190390.1
CS	F: ATCCTCCTAGAGCAGGGCAA<br>R: TGTGCTCATGGACTTGGGTC	204	XM_004006584.4
SIRT2	F: GCCAGACTGACCCTTTCCTC<br>R: GGTGGTGGAGAATTCCTGGG	253	XM_027978260.1
FADS1	F:GCTGCCCAATCTGAGCAAAGC<br>R:TCCTGTCATGGTGTGGCTCCTG	200	XM_004019593.4
PNPLA2	F:CGCCTAGGCATCTCTCTGAC<br>R:TCATAGAGCGGCAGGTTGTC	194	NM_001308576.1
APOA1	F:CTGAGGCAGGAGATGCACAA<br>R:CTCGTGCCACTTCTTCTGGA	87	U94720.1
ACSL3	F:AGCTGTCCCGGAAATCATGG<br>R:TTTCCACCTAGCAAGCCTCG	189	XM_027965239.1
ACAT1	F:AAGGTATGCGGAGCGAAGTT<br>R:ACCAAGTTTAGTGGCTGGCA	127	XM_027979167.1

Development • Supplementary information

SI References

1. P. Holm, P. J. Booth, M. H. Schmidt, T. Greve, H. Callesen, High bovine blastocyst development in a static in vitro production system using SOFaa medium supplemented with sodium citrate and myo-inositol with or without serum-proteins. *Theriogenology* **52**, 683-700 (1999).
2. P. Ramos-Ibeas *et al.*, Embryonic disc formation following post-hatching bovine embryo development in vitro. *Reproduction* **160**, 579-589 (2020).
3. A. Deglincerti *et al.*, Self-organization of the in vitro attached human embryo. *Nature* **533**, 251-254 (2016).
4. J. Schindelin *et al.*, Fiji: an open-source platform for biological-image analysis. *Nat Methods* **9**, 676-682 (2012).
5. P. Bermejo-Alvarez, R. M. Roberts, C. S. Rosenfeld, Effect of glucose concentration during in vitro culture of mouse embryos on development to blastocyst, success of embryo transfer, and litter sex ratio. *Mol Reprod Dev* **79**, 329-336 (2012).
6. P. Bermejo-Alvarez, D. Rizos, P. Lonergan, A. Gutierrez-Adan, Transcriptional sexual dimorphism during preimplantation embryo development and its consequences for developmental competence and adult health and disease. *Reproduction* **141**, 563-570 (2011).
7. P. Bermejo-Alvarez, D. Rizos, D. Rath, P. Lonergan, A. Gutierrez-Adan, Sex determines the expression level of one third of the actively expressed genes in bovine blastocysts. *Proc Natl Acad Sci U S A* **107**, 3394-3399 (2010).
8. T. D. Schmittgen, K. J. Livak, Analyzing real-time PCR data by the comparative C(T) method. *Nature protocols* **3**, 1101-1108 (2008).
9. S. Andrews (2017) FastQC: a quality control tool for high throughput sequence data. 2010.
10. S. Chen, Y. Zhou, Y. Chen, J. Gu, fastp: an ultra-fast all-in-one FASTQ preprocessor. *Bioinformatics* **34**, i884-i890 (2018).
11. N. L. Bray, H. Pimentel, P. Melsted, L. Pachter, Near-optimal probabilistic RNA-seq quantification. *Nat Biotechnol* **34**, 525-527 (2016).
12. M. I. Love, W. Huber, S. Anders, Moderated estimation of fold change and dispersion for RNA-seq data with DESeq2. *Genome Biol* **15**, 550 (2014).
13. C. Soneson, M. I. Love, M. D. Robinson, Differential analyses for RNA-seq: transcript-level estimates improve gene-level inferences. *F1000Res* **4**, 1521 (2015).
14. S. Durinck, P. T. Spellman, E. Birney, W. Huber, Mapping identifiers for the integration of genomic datasets with the R/Bioconductor package biomaRt. *Nat Protoc* **4**, 1184-1191 (2009).
15. C. P. Cantalapiedra, A. Hernández-Plaza, I. Letunic, P. Bork, J. Huerta-Cepas, eggNOG-mapper v2: Functional Annotation, Orthology Assignments, and Domain Prediction at the Metagenomic Scale. *Mol Biol Evol* (2021).
16. J. Huerta-Cepas *et al.*, eggNOG 5.0: a hierarchical, functionally and phylogenetically annotated orthology resource based on 5090 organisms and 2502 viruses. *Nucleic Acids Res* **47**, D309-D314 (2019).
17. B. Buchfink, C. Xie, D. H. Huson, Fast and sensitive protein alignment using DIAMOND. *Nat Methods* **12**, 59-60 (2015).

Development • Supplementary information
