# Supplementary material for: In vitro culture of ovine embryos up to early gastrulating stages
Source: Development. 2022 Mar 24;149(6):dev199743. doi: 10.1242/dev.199743 (PMC8977095; doi:10.1242/dev.199743)
Supplement: Reviewer comments [file develop-149-199743_review_history.pdf]

In vitro culture of ovine embryos up to early gastrulating stages

Priscila Ramos-Ibeas, Leopoldo González-Brusi, María Torres-Used, María Jesús Cocero, Pilar Marigorta, Ramiro Alberio and Pablo Bermejo-Álvarez
DOI: 10.1242/dev.199743

Editor: Maria Elena Torres-Padilla

Review timeline

Original submission:	26 April 2021
Editorial decision:	21 May 2021
First revision received:	29 November 2021
Editorial decision:	25 January 2022
Second revision received:	2 February 2022
Accepted:	3 February 2022

Original submission

First decision letter

MS ID#: DEVELOP/2021/199743

MS TITLE: In vitro culture of ovine embryos up to gastrulating stages

AUTHORS: Priscila Ramos-Ibeas, Maria Torres-Used, Maria Jesus Cocero, Pilar Marigorta, Ramiro Alberio, and Pablo Bermejo-Alvarez

I have now received all the referees' reports on the above manuscript, and have reached a decision. The referees' comments are appended below, or you can access them online: please go to BenchPress and click on the 'Manuscripts with Decisions' queue in the Author Area.

As you will see, the referees express considerable interest in your work, but have some significant criticisms and recommend a substantial revision of your manuscript before we can consider publication. In brief, the reviewers suggest additional characterisation of the structures developed in culture to ascertain their developmental identity. Also, the reviewers raised concerns on some overstatement in the degree of morphology and developmental timing of the culture system, compared to in vivo developed embryos. An important point is the degree to which gastrulation occurs in these structures, which would require additional characterisation, as suggested by all reviewers.

In addition to the points above, I would like to suggest a more in-depth transcriptomics analysis as suggested by Reviewer 2. While I recognise that this may require significant effort, and pose problems in terms of genome annotation, if you would be in the position to provide these data, the manuscript would be greatly strengthened. Alternatively, if scRNAseq is not feasible, some degree of molecular characterisation would be necessary instead, along the lines suggested by the reviewers.

If you are able to revise the manuscript along the lines suggested, which may involve further experiments, I will be happy receive a revised version of the manuscript. Your revised paper will be re-reviewed by one or more of the original referees, and acceptance of your manuscript will depend on your addressing satisfactorily the reviewers' major concerns. Please also note that Development will normally permit only one round of major revision.

© 2022. Published by The Company of Biologists under the terms of the Creative Commons Attribution License (https://creativecommons.org/licenses/by/4.0/).

We are aware that you may be experiencing disruption to the normal running of your lab that make experimental revisions challenging. If it would be helpful, we encourage you to contact us to discuss your revision in greater detail. Please send us a point-by-point response indicating where you are able to address concerns raised (either experimentally or by changes to the text) and where you will not be able to do so within the normal timeframe of a revision. We will then provide further guidance. Please also note that we are happy to extend revision timeframes as necessary.

Please attend to all of the reviewers' comments and ensure that you clearly highlight all changes made in the revised manuscript. Please avoid using 'Tracked changes' in Word files as these are lost in PDF conversion. I should be grateful if you would also provide a point-by-point response detailing how you have dealt with the points raised by the reviewers in the 'Response to Reviewers' box. If you do not agree with any of their criticisms or suggestions please explain clearly why this is so.

Reviewer 1

Advance summary and potential significance to field

The authors report on an advancement made to the culture of ruminant embryos, extending the time of development by several days. This is of value as the ruminant embryonic disk may be a much better model than the mouse egg cylinder. While this is an advancement, the authors should make clear that the in vitro D14 embryos are delayed in development as they are making a direct comparison to E11 or E12. It might help if the authors described the time equivalents between in vitro and in vivo embryos since the method of counting days for IVP produced and in vivo derived embryos can differ. That is, time-wise based on approximate time of fertilization, how do these compare?
While significant improvements in post-implantation culture in the mouse may have been made in recent years, pioneering work in the 1970 and 80s shouldn't be totally discounted [line 59]. There were a considerable number of reports of what were then called "outgrowths" in the 1970s and 90s. In those studies mouse embryos were able form two layer egg-cylinders under in vitro conditions. See A. Spindle. In Vitro 1980. An improved culture medium for mouse blastocysts. See also Gonda and Hsu. 1980 and Wiley & Pedersen
(1977).

Comments for the author

The data supports the authors' conclusions that the period of culture has been extended and will prove useful to those in the field. However, as the embryos remained spheroid, the authors have not yet reached the elusive goal of obtaining elongation in vitro. This must be acknowledged upfront.
Line 201 Should mention the delay in development time-wise Minor Line 48 Revise - the mammalian blastocysts is comprised of three different lineages……… form…… form….
Figure3. Image is missing 3F) or at least the label for F. The set of images in the lower left corner are presumably D and F but are not clearly identified.
Abstract:
Suggest that you use the phrase hatching from the zona pellucida or escaping from the zona pellucida, not just hatching.

Reviewer 2

Advance summary and potential significance to field

The authors set out a novel system for culturing ovine embryos from blastocyst stages to early gastrulation in vitro. They describe key features of early ovine development in these in vitro cultured embryos, including the formation of an embryonic disc, the maturation of the trophoblast and hypoblast, the disappearance of Rauber's layer and early stages of gastrulation.

This system represents an advance on current methods of ovine embryo culture which have focussed primarily on the development and morphogenesis of the extraembryonic tissues. Other

© 2022. Published by The Company of Biologists under the terms of the Creative Commons Attribution License (https://creativecommons.org/licenses/by/4.0/).

approaches for studying in vitro derived or cultured embryos have relied on reintroducing the embryo to the uterine environment for a period before recovering the embryo once more. The present study therefore makes the formation and development of the epiblast accessible to experimental investigation in vitro and will reduce the requirement for recipient female animals as a consequence (an advantage for the 3Rs principles). As such, it opens up significant opportunities for insight into the development of an organism that has hitherto been understudied - with implications for comparative development and evo-devo studies, technological in vitro culture system improvements, and the potential to apply knowledge gained to the development of other species in the future.

Overall, this paper is a careful and detailed first description of ovine embryo culture methods from blastocyst to early gastrula stage wholly in vitro. It is recommended for publication as a Techniques and Resources Article, provided that the issues below are addressed and minor corrections are made to the text and figures.

Comments for the author

Ramos-Ibeas et al., 2021

Summary of Advance & Significance:

The authors set out a novel system for culturing ovine embryos from blastocyst stages to early gastrulation *in vitro*. They describe key features of early ovine development in these *in vitro* cultured embryos, including the formation of an embryonic disc, the maturation of the trophoblast and hypoblast, the disappearance of Rauber's layer and early stages of gastrulation.

This system represents an advance on current methods of ovine embryo culture, which have focussed primarily on the development and morphogenesis of the extraembryonic tissues. Other approaches for studying *in vitro* derived or cultured embryos have relied on reintroducing the embryo to the uterine environment for a period before recovering the embryo once more. The present study therefore makes the formation and development of the epiblast accessible to experimental investigation *in vitro* and will reduce the requirement for recipient female animals as a consequence (an advantage for the 3Rs principles). As such, it opens up significant opportunities for insight into the development of an organism that has hitherto been understudied - with implications for comparative development and evo-devo studies, technological *in vitro* culture system improvements, and the potential to apply knowledge gained to the development of other species in the future.

Overall, this paper is a careful and detailed first description of ovine embryo culture methods from blastocyst to early gastrula stage wholly *in vitro*. It is recommended for publication as a Techniques and Resources Article, provided that the issues below are addressed and minor corrections are made to the text and figures.

Suggestions to Authors - Issues that must be addressed:

L33-34 (abstract), L181, L280, Figure 4 (caption), Figure S6 (caption)

One of the main claims of the manuscript is that the *in vitro* embryos are gastrulating. To support this claim, the authors should provide further evidence that the T+ cells are mesodermal, undergoing EMT, and that the primitive streak morphological structure can be identified in the *in vitro* cultured embryos.

In a previously published article, Guillomot *et al.* (2004) show that *Brachyury* is detectable in one pole of the epiblast and subsequently throughout the primitive streak of ovine embryos, in a manner similar to mouse development where *Brachyury* is detectable only in the mesoderm directly adjacent to the streak at early-streak and mid-streak stages. As such, Brachyury expression is transient and marks the region of the site of gastrulation, rather than the successful completion of gastrulation itself. The authors should therefore analyse the expression of a later mesodermal marker in the *in vitro*-cultured embryos if they are to substantiate the claim that the dispersed T+ cells are truly mesodermal. Analysing the expression of EMT-specific genes such as SNAIL or N-CADH in these cells would also

© 2022. Published by The Company of Biologists under the terms of the Creative Commons Attribution License (https://creativecommons.org/licenses/by/4.0/).

strengthen the argument that the dispersed cells are migratory and have ingressed through the streak. It would also be appropriate to re-examine the Laminin stainings shown in Figure 4A and S5 for any signs of basement membrane breakdown, indicating an area associated with EMT.

Methods

It is not clear from the text how the quantification data were collected. Namely, the data from the embryo survival, ED formation, Hypoblast migration etc. Was this done by-eye or using software of some sort? Was it manual or automatic? If manual, then ideally these should have been blinded and different users allocated subsections of the data (then tested for user-differences). The authors should then also provide evidence of representative examples from each category. If (semi-)automatic, then the software used and any parameters need to be fully described. As it currently reads, it is impossible to know how these data were drawn and whether any human error or bias might have contributed to the effects observed. Since the strength of the conclusions drawn relies almost entirely on these quantifications, this is important to describe and account for.

L143-144, L157-159, L111-113 etc
In various areas of the text, the authors report ‘trends’ in data that are not statistically significant. This should be avoided, as non-significant differences should be reported as no differences at all.

Minor Revisions:

L60 – Suggest the inclusion of reference to Hsu (1979)[1] as a culture system supporting the development of mouse embryos beyond implantation.

L96-98, Figure 1A – Please clarify the duration of the culture in the different media in the main text. The SI Materials & Methods describes this period as being from D6/D7 after IVF to D14 with medium changes as described.

L101, Table 1 – Please clarify in the Materials & Methods how “epiblast survival” was scored (e.g. any remaining SOX2+ cell(s) at D14).

L143-144 - Amend reporting of the comparison of SOX2+ cell numbers between N2B27+A+R (~74) and N2B27+A (~41) or N2B27+R (~60), which is described as “slightly higher.” An appropriate statistical test should be conducted to determine whether this difference is indeed significant.

Figure 2B, Figure 2E, Figure 3E – For embryos grown in the presence of ROCK inhibitor (N2B27+R; N2B27+A+R), there appears to be a proportion of embryos with high numbers of SOX2+ cells (>300). The authors are advised to investigate the robustness of their statistical tests with bootstrapping in these cases (to examine the dependence on outliers) and to report their findings in the Statistical Analysis section of the Supplementary Information.

Figure 2B – Consider changing the representation of the data to better display the distribution at low numbers of SOX2+ cells (<100). We suggest including a metric/graph of the proportion of embryos in each condition with zero SOX2+ cells.

Figure 2C - It would assist the reader to include (or be referred to) an image of an *in vivo* control at D14, for comparison with the images of *in vitro* cultured embryos.

[1]Hsu YC: In vitro development of individually cultured whole mouse embryos from blastocyst to early somite stage. *Dev Biol* 1979, 68:453–461.

© 2022. Published by The Company of Biologists under the terms of the Creative Commons Attribution License (https://creativecommons.org/licenses/by/4.0/).

Figure 3B - Consider visualising the data on embryonic disc size with a histogram to capture the variability in these data rather than just the mean. Currently, single points do not allow the reader to adequately assess the variability in the data.

Figure 4B, Figure S5 - Please note the identity of the layer of cells underlying the epiblast in the E11 *in vivo* embryos, if known. This appears to be absent from the D14 *in vitro* cultured embryos and may be worthy of comment.

L183 - The location of the T+ cells is described as the posterior of the ED, as defined by T expression. Without another posterior marker, this argument is circular. This expression could instead be referred to as polarised/polar/asymmetric in the absence of another marker (e.g. CDX) or a visible anatomical landmark like the primitive streak.

L191 - Were any binucleate cells observed prior to D14 in the *in vitro* embryos? The *in vivo* data would suggest that they would not be present at earlier stages; it would be worth noting if they appear at an expected point in time.

L251-254 - Consider including a similar statement to summarise the ED formation and variability in SOX2+ cell number in *in vitro* cultured embryos. Does the level of variability seem comparable between the *in vitro* cultured embryos and those that have developed *in vivo?*

Supplementary Information (Immunofluorescence) - In counting the number of SOX2+ or TUNEL+ cells, consider adding a note describing whether these quantifications were blinded at all, or whether they were conducted by different users.

Figure S2 & S3 - These figures provide a helpful illustration of the lack of Rauber's layer in an *in vitro* cultured embryo grown in N2B27+A+R, in comparison to an embryo cultured in N2B27 alone. The absence of trophoblast cells over the epiblast would be made clearer by using an alternative lookup table for the DAPI channel (such as grey, orange or magenta). The arrowheads in Figure S3 are helpful in this regard.

Typographical changes:

L102, L126, L137, L139, L144, L148, L156, L160, L161, L166-168, L172, L181, L192, L269 - When reporting approximate numerical data, specify the mean and express the variability as a standard deviation, instead of using a tilde and rounded approximation.

L164 - Consider amending reference to Fig. 3D-F to Fig. 3E.

Methods - although details are in the Supplementary text, the information about the age of the blastocysts at the start of the in vitro culture method is not in the main text, and should be added.

Figure 2A - Amend typo in y-axis label: "cellls."

Figure 2 Caption - Amend the figure labelling to reflect the order of the panels B and C.

Table 2 Caption - "No differences were found in … survival" - Please clarify that this refers to embryonic survival, rather than the separate epiblast survival metric. The significant difference between these culture conditions appears to be in epiblast survival.

Table S3 - Correct typo in units of Fragment size ("bp" not "pb").

Figure S1 - Update panel labelling (A and B) for consistency with the figure caption.

Figure S6 (caption) - Please clarify whether (A) and (B) represent the sections from two *in vitro* cultured embryos, or whether (B) is in fact an *in vivo* cultured specimen.

© 2022. Published by The Company of Biologists under the terms of the Creative Commons Attribution License (https://creativecommons.org/licenses/by/4.0/).

Reviewer 3

Advance summary and potential significance to field

The manuscript by Ramos-Ibeas and colleagues entitled “In vitro culture of ovine embryos up to gastrulating stages” provides an interesting way to get gastrulating ovine embryos from a novel in vitro culture system. They developed a new culture medium using N2/B27 supplements combined with ROCK inhibitor and activing A and used culture dish coated with agarose to avoid embryos' attachment to the dish. Using this system, they were able to produce gastrulating ovine embryos from an exclusively in vitro system.
The manuscript is of interest for a large panel of developmental biologist working on early embryonic development but also for those working on livestock reproduction and reproductive technology.

Comments for the author

The manuscript is well written and well documented. The characterization of the in vitro system and the resulting embryos is really well done and detailed. The difficulty of accessing good antibodies working in the ovine species may have limited the characterization of the different embryonic tissues and a single marker was used for each tissue: SOX17 for the hypoblast, SOX2 for the epiblast and GATA3 for the trophoblast.
The molecular characterization of gastrulation remains insufficient even though the data strongly suggest that it occurs. Thus, the T labelling is informative but should be completed by a better description of the formation of the mesendoderm and of the epithelial-mesenchymal transition (EMT). It would be useful to look at the expression of known markers of the primitive streak like SNAI1 and NODAL, but also to describe the loss of the basal lamina, the disappearance of adherent and tight junctions (loss of E-cadherin, ZO1).
Likewise, the trophoblast is poorly characterized and the image from FIG. 4D shows a certain disorganization of the epithelium in comparison with the embryo at E12.5 (Fig. 4E). The authors should comment and clarify this point. In addition, do they observe mural trophoblast around the ED?
Finally, a comparative transcriptomic analysis between the D14, E11 and E12.5 embryos is necessary to visualize the molecular proximity of the embryos produced in vitro and in vivo. A scRNA-seq analysis on the 3 stages would be even more relevant to demonstrate the coexistence of the different cell populations in embryos produced in vitro compared to embryos obtained in vivo.

Minor points:
Line 140: should be Fig.2C instead of Fig. 2D.
Line 207: D14 embryos are in between E11 and E12.5 embryos, but do not really reflect the E12.5 stage. This is not clear from the sentence.

First revision

Author response to reviewers' comments

Editor comments

Dear Dr. Ramos-Ibeas,

I have now received all the referees' reports on the above manuscript, and have reached a decision. The referees' comments are appended below, or you can access them online: please go to BenchPress and click on the 'Manuscripts with Decisions' queue in the Author Area.

As you will see, the referees express considerable interest in your work, but have some significant criticisms and recommend a substantial revision of your manuscript before we can consider publication. In brief, the reviewers suggest additional characterisation of the structures developed in culture to ascertain their developmental identity. Also, the reviewers raised concerns on some

© 2022. Published by The Company of Biologists under the terms of the Creative Commons Attribution License (https://creativecommons.org/licenses/by/4.0/).

overstatement in the degree of morphology and developmental timing of the culture system, compared to in vivo developed embryos. An important point is the degree to which gastrulation occurs in these structures, which would require additional characterisation, as suggested by all reviewers.

In addition to the points above, I would like to suggest a more in-depth transcriptomics analysis as suggested by Reviewer 2. While I recognise that this may require significant effort, and pose problems in terms of genome annotation, if you would be in the position to provide these data, the manuscript would be greatly strengthened. Alternatively, if scRNAseq is not feasible, some degree of molecular characterisation would be necessary instead, along the lines suggested by the reviewers.

We thank the editor and reviewers for their time and helpful comments. We also apologize for the delayed response, *in vivo* experiments in large animals are time consuming. We have conducted the additional experiments and changes requested, which we believe have resulted in an improved and easier to follow manuscript. The revised version includes additional characterization of the structures, particularly focusing on the *in vitro* gastrulating embryonic discs. We have also included additional markers of developmental landmarks of gastrulation and performed RNAseq analysis of *in vitro* and *in vivo* embryos at two developmental times.

Reviewer 1 Advance Summary and Potential Significance to Field...

The authors report on an advancement made to the culture of ruminant embryos, extending the time of development by several days. This is of value as the ruminant embryonic disk may be a much better model than the mouse egg cylinder. While this is an advancement, the authors should make clear that the in vitro D14 embryos are delayed in development as they are making a direct comparison to E11 or E12. It might help if the authors described the time equivalents between in vitro and in vivo embryos since the method of counting days for IVP produced and in vivo derived embryos can differ. That is, time-wise based on approximate time of fertilization, how do these compare?

We thank the interest of the reviewer in ruminant embryos as a close model of human gastrulation. We agree that, as occurring for pre-hatching conventional culture, post-hatching *in vitro* culture entails a clear delay in embryo development. We have now highlighted such delay and provided an estimation based on the developmental landmarks, transcription and appearance of *in vitro* embryos on lines 228-233. Regarding the equivalence in developmental timing between *in vivo* and *in vitro*, it is certainly difficult to find a perfect match reflecting the moment of fertilization (i.e. sperm penetration) on both systems. We decided to use the conventional starting points applied in previous literature (i.e. start of gamete co-incubation for *in vitro* “D” or mating for *in vivo* “E”) because they almost coincide on time and provide an easy comparison with previous literature: *in vitro* fertilization is expected to occur at ~D0.5 while *in vivo* fertilization may occur from E0.5 to E1. To clarify *in vivo* and *in vitro* timing, we expanded Fig. S9 (former S7) including the *in vitro* group.

While significant improvements in post-implantation culture in the mouse may have been made in recent years, pioneering work in the 1970 and 80s shouldn’t be totally discounted [line 59]. There were a considerable number of reports of what were then called “outgrowths” in the 1970s and 90s. In those studies mouse embryos were able form two layer egg-cylinders under in vitro conditions. See A. Spindle. In Vitro 1980. An improved culture medium for mouse blastocysts. See also Gonda and Hsu. 1980 and Wiley & Pedersen (1977).

We have added these references to the introduction (lines 59-61).

Reviewer 1 Comments for the Author...

The data supports the authors’ conclusions that the period of culture has been extended and will prove useful to those in the field. However, as the embryos remained spheroid, the authors have not yet reached the elusive goal of obtaining elongation in vitro. This must be acknowledged upfront.

© 2022. Published by The Company of Biologists under the terms of the Creative Commons Attribution License (https://creativecommons.org/licenses/by/4.0/).

This fact is now acknowledged in multiple parts of the manuscript (lines 101, 155, 208, 229, 278 and 332-333).

Line 201 Should mention the delay in development time-wise
The developmental delay has been now highlighted and explained on lines 228-233.

Minor
Line 48 Revise - the mammalian blastocysts is comprised of three different lineages……… form…… form….
This has been amended, thank you for the correction.

Figure3. Image is missing 3F) or at least the label for F. The set of images in the lower left corner are presumably D and F but are not clearly identified.
This has been amended, thank you for the correction.

Abstract:
Suggest that you use the phrase hatching from the zona pellucida or escaping from the zona pellucida, not just hatching.
This has been amended, thank you for the suggestion.

Reviewer 2
Summary of Advance & Significance:
The authors set out a novel system for culturing ovine embryos from blastocyst stages to early gastrulation *in vitro*. They describe key features of early ovine development in these *in vitro* cultured embryos, including the formation of an embryonic disc, the maturation of the trophoblast and hypoblast, the disappearance of Rauber's layer and early stages of gastrulation.

This system represents an advance on current methods of ovine embryo culture, which have focused primarily on the development and morphogenesis of the extraembryonic tissues.
Other approaches for studying *in vitro* derived or cultured embryos have relied on reintroducing the embryo to the uterine environment for a period before recovering the embryo once more. The present study therefore makes the formation and development of the epiblast accessible to experimental investigation *in vitro* and will reduce the requirement for recipient female animals as a consequence (an advantage for the 3Rs principles). As such, it opens up significant opportunities for insight into the development of an organism that has hitherto been understudied - with implications for comparative development and evo-devo studies, technological *in vitro* culture system improvements, and the potential to apply knowledge gained to the development of other species in the future.
Overall, this paper is a careful and detailed first description of ovine embryo culture methods from blastocyst to early gastrula stage wholly *in vitro*. It is recommended for publication as a Techniques and Resources Article, provided that the issues below are addressed and minor corrections are made to the text and figures.

We thank the reviewer for numbering wide applications of the system and model from different perspectives of development.

Suggestions to Authors - Issues that must be addressed:

L33-34 (abstract), L181, L280, Figure 4 (caption), Figure S6 (caption)
One of the main claims of the manuscript is that the *in vitro* embryos are gastrulating. To support this claim, the authors should provide further evidence that the T+ cells are mesodermal, undergoing EMT, and that the primitive streak morphological structure can be identified in the *in vitro* cultured embryos.
In a previously published article, Guillomot *et al.* (2004) show that *Brachyury* is detectable in one pole of the epiblast and subsequently throughout the primitive streak of ovine embryos, in a manner similar to mouse development where *Brachyury* is detectable only in the mesoderm directly adjacent to the streak at early-streak and mid-streak stages. As such, Brachyury expression is transient and marks the region of the site of gastrulation, rather than the successful completion of gastrulation itself. The authors should therefore analyse the expression of a later mesodermal marker in the *in vitro*-cultured embryos if they are to substantiate the claim that the

© 2022. Published by The Company of Biologists under the terms of the Creative Commons Attribution License (https://creativecommons.org/licenses/by/4.0/).

dispersed T+ cells are truly mesodermal. Analysing the expression of EMT-specific genes such as SNAIL or N-CADH in these cells would also strengthen the argument that the dispersed cells are migratory and have ingressed through the streak.

We have performed different additional experiments to detect markers for stable mesoderm, EMT transition and anterior-posterior patterning. Although we were not able to find a suitable antibody to detect SNAIL in sheep cells, we detected N-cadherin positive cells undergoing EMT in 44.4% D14 *in vitro* embryos containing an ED (n=4/9) (lines 184-190 and Fig4D) and in all E12.5 *in vivo* embryos. We have also analysed HESX1 expression with an antibody reported to label the anterior part of the embryonic disc at the onset of gastrulation in human embryos (Xiang et al., 2020). However, only hypoblast cells stained positive for HESX1 in the sheep embryo (lines 194-199 and Fig S8). Finally, we also provide immunostainings for EOMES, another protein playing a major role in primitive streak formation, which colocalizes with T+ cells in *in vivo* and *in vitro* embryos (lines 190-193 and Fig 4E).

It would also be appropriate to re-examine the Laminin stainings shown in Figure 4A and S5 for any signs of basement membrane breakdown, indicating an area associated with EMT.

We have re-examined Laminin stainings (7 embryos) but while some breaking could be inferred on some pictures, we had not enough resolution in whole-mount embryos to identify a clear basement membrane breakdown.

Methods

It is not clear from the text how the quantification data were collected. Namely, the data from the embryo survival, ED formation, Hypoblast migration etc. Was this done by-eye or using software of some sort? Was it manual or automatic? If manual, then ideally these should have been blinded and different users allocated subsections of the data (then tested for user differences).

The authors should then also provide evidence of representative examples from each category. If (semi-)automatic, then the software used and any parameters need to be fully described. As it currently reads, it is impossible to know how these data were drawn and whether any human error or bias might have contributed to the effects observed. Since the strength of the conclusions drawn relies almost entirely on these quantifications, this is important to describe and account for.

We apologize for not describing clearly how developmental parameters were evaluated. As shown in Fig. S10, developmental parameters are easily identifiable and not prone to a subjective bias. The criteria used to identify embryo survival, ED formation, epiblast survival and epiblast migration is now explained in SI materials and methods and on Fig. S10 legend.

L143-144, L157-159, L111-113 etc

In various areas of the text, the authors report ‘trends’ in data that are not statistically significant. This should be avoided, as non-significant differences should be reported as no differences at all.

We agree with the reviewer and have modified the text accordingly.

Minor Revisions:

L60 – Suggest the inclusion of reference to Hsu (1979) as a culture system supporting the development of mouse embryos beyond implantation.

We have added this reference to line 60.

L96-98, Figure 1A – Please clarify the duration of the culture in the different media in the main text. The SI Materials & Methods describes this period as being from D6/D7 after IVF to D14 with medium changes as described.

We have added this information to line 95.

L101, Table 1 – Please clarify in the Materials & Methods how “epiblast survival” was scored (e.g. any remaining SOX2+ cell(s) at D14).

We have added this information to Supplementary materials & methods.

© 2022. Published by The Company of Biologists under the terms of the Creative Commons Attribution License (https://creativecommons.org/licenses/by/4.0/).

L143-144 - Amend reporting of the comparison of SOX2+ cell numbers between N2B27+A+R (~74) and N2B27+A (~41) or N2B27+R (~60), which is described as "slightly higher." An appropriate statistical test should be conducted to determine whether this difference is indeed significant.
We have modified the text (lines 145-147) to remove this information because no statistically significant differences were found between these parameters.

Figure 2B, Figure 2E, Figure 3E - For embryos grown in the presence of ROCK inhibitor (N2B27+R; N2B27+A+R), there appears to be a proportion of embryos with high numbers of SOX2+ cells (>300). The authors are advised to investigate the robustness of their statistical tests with bootstrapping in these cases (to examine the dependence on outliers) and to report their findings in the Statistical Analysis section of the Supplementary Information.
We have performed non-parametric bootstrapping with 1000 replicates per statistical test, and dependence on outliers was discarded. We report these findings in Supplementary materials & methods.

Figure 2B - Consider changing the representation of the data to better display the distribution at low numbers of SOX2+ cells (<100). We suggest including a metric/graph of the proportion of embryos in each condition with zero SOX2+ cells.
We have modified the graphs in Fig 2B and 2E to better display the embryos with less than 100 SOX2+ cells. The proportion of embryos without SOX2+ cells can be inferred from the information in Table 2 - epiblast survival (% of embryos showing any SOX2+ cells).

Figure 2C - It would assist the reader to include (or be referred to) an image of an *in vivo* control at D14, for comparison with the images of *in vitro* cultured embryos.
We have now included E14 *in vivo* embryo images in Fig 3.

Figure 3B - Consider visualising the data on embryonic disc size with a histogram to capture the variability in these data rather than just the mean. Currently, single points do not allow the reader to adequately assess the variability in the data.
We have now represented these data as histograms including dots. We have also included E14 *in vivo* data and embryo length is represented instead of embryo area because E14 embryo area could not be accurately measured due to their irregular morphology.

Figure 4B, Figure S5 - Please note the identity of the layer of cells underlying the epiblast in the E11 *in vivo* embryos, if known. This appears to be absent from the D14 *in vitro* cultured embryos and may be worthy of comment.
We have now indicated in these Figures that this layer of cells is the hypoblast.
These cells were also present in D14 *in vitro* embryos, although their staining intensity in the embryo shown was lower. Since this embryo had the most representative aPKC staining, we have increased the brightness of hypoblast cells without overexposing epiblast cells, which form a much more compact and bright structure. We apologize for this inconvenient; images in this work are taken from whole embryos and some of them are difficult to capture in the perfect orientation.

L183 - The location of the T+ cells is described as the posterior of the ED, as defined by T expression. Without another posterior marker, this argument is circular. This expression could instead be referred to as polarised/polar/asymmetric in the absence of another marker (e.g. CDX) or a visible anatomical landmark like the primitive streak.
Thank you for this suggestion. We provide immunostainings for EOMES, another protein playing a major role in primitive streak formation. We show co-expression with T+ cells in *in vivo* and *in vitro* embryos (lines 190-193 and Fig 4E). We consider it unlikely both EOMES and T being anterior markers in sheep, in contrast to humans or mice.
We also stained for HESX1, which labelled the anterior part of the human embryonic disc at the onset of gastrulation (Xiang et al., 2020), but unfortunately it was hypoblast specific in sheep embryos (Fig S8; *in vivo* embryo).

L191 - Were any binucleate cells observed prior to D14 in the *in vitro* embryos? The *in vivo* data would suggest that they would not be present at earlier stages; it would be worth noting if they appear at an expected point in time.
We have now analyzed D12 *in vitro* embryos (n=23), and did not find any binucleate cells at this

© 2022. Published by The Company of Biologists under the terms of the Creative Commons Attribution License (https://creativecommons.org/licenses/by/4.0/).

stage (lines 204-205).

L251-254 – Consider including a similar statement to summarise the ED formation and variability in SOX2+ cell number in *in vitro* cultured embryos. Does the level of variability seem comparable between the *in vitro* cultured embryos and those that have developed *in vivo?*
We have now discussed ED formation and variability in *in vitro* embryos, which was similar to that obtained in E12.5 *in vivo* embryos. This variability was not observed at E14 *in vivo*, presumably because embryos lacking an ED had already degenerated (lines 281-287).

Supplementary Information (Immunofluorescence) - In counting the number of SOX2+ or TUNEL+ cells, consider adding a note describing whether these quantifications were blinded at all, or whether they were conducted by different users.
We have now included this information in supplementary materials and methods under "Data and statistical analysis".

Figure S2 & S3 – These figures provide a helpful illustration of the lack of Rauber's layer in an *in vitro* cultured embryo grown in N2B27+A+R, in comparison to an embryo cultured in N2B27 alone. The absence of trophoblast cells over the epiblast would be made clearer by using an alternative lookup table for the DAPI channel (such as grey, orange or magenta).
The arrowheads in Figure S3 are helpful in this regard.
We have now used grey lookup table for Fig 2D, S2, S3 and S4 to better visualize trophoblast cells. Thank you for the advice.

Typographical changes:
L102, L126, L137, L139, L144, L148, L156, L160, L161, L166-168, L172, L181, L192, L269 - When reporting approximate numerical data, specify the mean and express the variability as a standard deviation, instead of using a tilde and rounded approximation.
Approximate numerical data have been replaced by the exact mean ± s.e.m.

L164 – Consider amending reference to Fig. 3D-F to Fig. 3E.
We have modified the reference, according to the revised Fig 3.

Methods - although details are in the Supplementary text, the information about the age of the blastocysts at the start of the in vitro culture method is not in the main text, and should be added.
We have added this information in the main materials and methods (line 358).

Figure 2A – Amend typo in y-axis label: "cellls."
We thank the reviewer for noticing this mistake, we have amended this typo.

Figure 2 Caption – Amend the figure labelling to reflect the order of the panels B and C. We have amended this mistake.

Table 2 Caption – "No differences were found in … survival" – Please clarify that this refers to embryonic survival, rather than the separate epiblast survival metric. The significant difference between these culture conditions appears to be in epiblast survival.
We have specified that this sentence refers to embryonic survival.

Table S3 - Correct typo in units of Fragment size ("bp" not "pb"). We have amended this mistake.

Figure S1 - Update panel labelling (A and B) for consistency with the figure caption. We have amended this mistake.

Figure S6 (caption) – Please clarify whether (A) and (B) represent the sections from two *in vitro* cultured embryos, or whether (B) is in fact an *in vivo* cultured specimen. We apologize for this mistake. We have now specified that (B) represents an *in vivo* embryo.

© 2022. Published by The Company of Biologists under the terms of the Creative Commons Attribution License (https://creativecommons.org/licenses/by/4.0/).

Reviewer 3 Advance Summary and Potential Significance to Field...

The manuscript by Ramos-Ibeas and colleagues entitled “In vitro culture of ovine embryos up to gastrulating stages” provides an interesting way to get gastrulating ovine embryos from a novel in vitro culture system. They developed a new culture medium using N2/B27 supplements combined with ROCK inhibitor and activing A and used culture dish coated with agarose to avoid embryos' attachment to the dish. Using this system, they were able to produce gastrulating ovine embryos from an exclusively in vitro system.
The manuscript is of interest for a large panel of developmental biologist working on early embryonic development but also for those working on livestock reproduction and reproductive technology.

We thank the interest shown in the system, which we hope will be also helpful to other groups working on diverse fields.

Reviewer 3 Comments for the Author...

The manuscript is well written and well documented. The characterization of the in vitro system and the resulting embryos is really well done and detailed. The difficulty of accessing good antibodies working in the ovine species may have limited the characterization of the different embryonic tissues and a single marker was used for each tissue: SOX17 for the hypoblast, SOX2 for the epiblast and GATA3 for the trophoblast.
We also consider it important to provide useful information on resources, particularly on specific antibodies. We have now added validation of the antibodies used with co-expressing proteins in Fig S10. In particular, SOX2 has been co-stained with NANOG; SOX17 with GATA6 and FOXA2; GATA3 with CDX2; and T with EOMES.

The molecular characterization of gastrulation remains insufficient even though the data strongly suggest that it occurs. Thus, the T labelling is informative but should be completed by a better description of the formation of the mesendoderm and of the epithelial-mesenchymal transition (EMT). It would be useful to look at the expression of known markers of the primitive streak like SNAI1 and NODAL, but also to describe the loss of the basal lamina, the disappearance of adherent and tight junctions (loss of E-cadherin, ZO1).
We have performed additional experiments to analyse the expression of additional markers of mesoderm, EMT transition and anterior-posterior patterning. Although we could not find antibodies against SNAI1 or NODAL proved to react in sheep cells, we have observed N-cadherin positive cells undergoing EMT in 44.4% D14 *in vitro* embryos containing an ED (n=4/9) (lines 184- 190 and Fig 4D) and in E12.5 *in vivo* embryos. We have also analysed HESX1 expression with an antibody reported to label the anterior part of the embryonic disc at the onset of gastrulation in human embryos (Xiang et al., 2020). However, only hypoblast cells stained positive for HESX1 in the sheep embryo (lines 194-199 and Fig S8).
Finally, we also provide new immunostainings for EOMES, another protein playing a major role in primitive streak formation, which co-stains T+ cells in *in vivo* and *in vitro* embryos (lines 190- 193 and Fig 4E).

Likewise, the trophoblast is poorly characterized and the image from FIG. 4D shows a certain disorganization of the epithelium in comparison with the embryo at E12.5 (Fig. 4E). The authors should comment and clarify this point. In addition, do they observe mural trophoblast around the ED?
We have modified this paragraph to clearly state that trophoblast cells from *in vitro* embryos do not proliferate at the same extent than *in vivo*. Trophoblast cells from *in vitro* embryos display a bigger cytoplasm and more sparse nuclei than *in vivo*, as it can be observed in Fig 3D vs. E (lines 206-209).
Mural trophoblast is only observed around the embryo surface in which the embryonic disc is absent. As reported in Fig 2D and lines 161-163, the polar trophoblast around the ED (Rauber´s layer) disappeared in 22/36 embyos with ED developed in N2B27+A+R.

Finally, a comparative transcriptomic analysis between the D14, E11 and E12.5 embryos is necessary to visualize the molecular proximity of the embryos produced in vitro and in vivo. A scRNA-seq analysis on the 3 stages would be even more relevant to demonstrate the coexistence of

© 2022. Published by The Company of Biologists under the terms of the Creative Commons Attribution License (https://creativecommons.org/licenses/by/4.0/).

the different cell populations in embryos produced in vitro compared to embryos obtained in vivo.
We now provide a RNA-seq analysis of D14 *in vitro,* E11 and E12.5 *in vivo* embryos. We report and discuss these findings in lines 209-220, 321-334 and Figure 6.

Minor points:
Line 140: should be Fig.2C instead of Fig. 2D.
This sentence refers to the ED from an embryo cultured in N2B27 in Fig 2D.

Line 207: D14 embryos are in between E11 and E12.5 embryos, but do not really reflect the E12.5 stage. This is not clear from the sentence.
We agree with the reviewer. We have added a sentence to lines 228-231 which reflects the similarities of D14 *in vitro* embryos with either *in vivo* stage.

References:

Holm, P. and Callesen, H. (1998). In vivo versus in vitro produced bovine ova: similarities and differences relevant for practical application. *Reproduction Nutrition Development* **38**, 579-594.
Xiang, L., Yin, Y., Zheng, Y., Ma, Y., Li, Y., Zhao, Z., Guo, J., Ai, Z., Niu, Y., Duan, K., et al. (2020). A developmental landscape of 3D-cultured human pre-gastrulation embryos. *Nature* **577**, 537-542.

Second decision letter

MS ID#: DEVELOP/2021/199743

MS TITLE: In vitro culture of ovine embryos up to gastrulating stages.

AUTHORS: Priscila Ramos-Ibeas, Leopoldo Gonzalez-Brusi, Maria Torres-Used, Maria Jesus Cocero, Pilar Marigorta, Ramiro Alberio, and Pablo Bermejo-Alvarez

Please excuse the excessive amount of time that it has taken me to get back to you with our Reviewers' feedback on your above revised manuscript. The Christmas period caused severe delays with the Reviewers. Thank you for your patience.

I have now received all the referees reports and I am happy to report that we could accept your manuscript in Development, since all the Reviewers are globally satisfied with your revision. There are only a few minor points, which I would encourage you to correct, as follows.
Reviewer 3 requests that the title of your manuscript be adapted, to avoid sending a misleading message. I agree with this comment and therefore it would be important to adapt the title of your manuscript. The Reviewer has made a specific recommendation, which you could follow, or alternatively, you could suggest one along those lines.

Reviewer 3 has also identified a few errors in the reference to the figures, which would be important to fix (Page 5, line 143 and Page 6, line 155). If you could attend to the request of Reviewer 2 to split the DE table, that would be advisable, but not necessary. Thus, in short, if you could provide a final revised manuscript in which you update at least i) the title, and the ii) two lines of text that I have pointed out at your convenience, we could proceed promptly with formal acceptance and production.

The referees' comments are appended below, or you can access them online: please go to BenchPress and click on the 'Manuscripts with Decisions' queue in the Author Area.

Please provide a short cover letter upon submission, to confirm that you have done (or argue otherwise) my suggestions above.

© 2022. Published by The Company of Biologists under the terms of the Creative Commons Attribution License (https://creativecommons.org/licenses/by/4.0/).

Reviewer 1

Advance summary and potential significance to field

The authors report on an advancement made to the culture of ruminant embryos extending the time of development by several days. This is of value as the ruminant embryonic disk may be a much better model than the mouse egg cylinder

Comments for the author

No further revisions suggested.

Reviewer 2

Advance summary and potential significance to field

This manuscript marks and important advance in our ability to culture ovine embryos right up to the point of early gastrulation in vitro. It represents an important technical advance for the community.

Comments for the author

This paper is much improved over the previous version, and the authors have address all of my comments and concerns. I particularly think the RNA-seq dataset, albeit at the bulk level (and therefore difficult to distinguish between proportions of cell types rather than cellular identity), adds to the confidence that their in vitro embryos are mirroring in vivo development closely. My only remaining, minor comment would be that it could be useful to distinguish between DEG that are upregulated or downregulated between conditions (eg Fig 6A), as this might reveal some more insight - although I note that the data is available in table form if readers were inclined to examine it more closely.

Reviewer 3

Advance summary and potential significance to field

The manuscript by Ramos-Ibeas and colleagues entitled “In vitro culture of ovine embryos up to gastrulating stages” provides an interesting way to get gastrulating ovine embryos from a novel in vitro culture system. They developed a new culture medium using N2/B27 supplements combined with ROCK inhibitor and activing A and used culture dish coated with agarose to avoid embryos' attachment to the dish. Using this system, they were able to produce gastrulating ovine embryos from an exclusively in vitro system.
The manuscript is of interest for a large panel of developmental biologist working on early embryonic development but also for those working on livestock reproduction and reproductive technology.

Comments for the author

The reviewer thanks the authors for all the improvements made in the manuscript and in the figures.
The revised version addressed the different concerns raised by the reviewer from the previous version.
One remaining problem is that the title is somehow misleading because in vitro cultured embryos only recapitulate some of the events corresponding to the gastrulating stages. The authors should therefore modify the title to make it closer to the reality of the model, something like "In vitro culture of ovine embryos up to stages recapitulating features of gastrulation". Similarly in Discussion, page 8 line 224-225 the conclusion has to be toned down.
Minor points:
Fig.S1A and Fig/1C are redundant and provide the same information. Fig.S1A can be removed.
Page 5 line 143: Fig2C instead of Fig.2D Page 6, line 155: (E11, E12.5 and E14)

© 2022. Published by The Company of Biologists under the terms of the Creative Commons Attribution License (https://creativecommons.org/licenses/by/4.0/).

Fig.S8 and lines 195 to 200: this result is of interest but is not really necessary and does not fit well in the text.

Second revision

Author response to reviewers' comments

Dear Dr. Ramos-Ibeas,

Please excuse the excessive amount of time that it has taken me to get back to you with our Reviewers' feedback on your above revised manuscript. The Christmas period caused severe delays with the Reviewers. Thank you for your patience.

I have now received all the referees reports and I am happy to report that we could accept your manuscript in Development, since all the Reviewers are globally satisfied with your revision. There are only a few minor points, which I would encourage you to correct, as follows. Reviewer 3 requests that the title of your manuscript be adapted, to avoid sending a misleading message. I agree with this comment and therefore it would be important to adapt the title of your manuscript. The Reviewer has made a specific recommendation, which you could follow, or alternatively, you could suggest one along those lines.

Reviewer 3 has also identified a few errors in the reference to the figures, which would be important to fix (Page 5, line 143 and Page 6, line 155). If you could attend to the request of Reviewer 2 to split the DE table, that would be advisable, but not necessary. Thus, in short, if you could provide a final revised manuscript in which you update at least i) the title, and the ii) two lines of text that I have pointed out at your convenience, we could proceed promptly with formal acceptance and production.

Please provide a short cover letter upon submission, to confirm that you have done (or argue otherwise) my suggestions above.

We thank the editor and reviewers for their time and helpful final comments. We have followed the suggestions and corrected the errors identified by Reviewer 3 and we have modified Figure 6A according to the suggestions of Reviewer 2. Up-regulated genes in each condition are indicated with different colors in Table S2.

Reviewer 1 Advance Summary and Potential Significance to Field...

The authors report on an advancement made to the culture of ruminant embryos, extending the time of development by several days. This is of value as the ruminant embryonic disk may be a much better model than the mouse egg cylinder

Reviewer 1 Comments for the Author...

No further revisions suggested.

Reviewer 2 Advance Summary and Potential Significance to Field...

This manuscript marks and important advance in our ability to culture ovine embryos right up to the point of early gastrulation in vitro. It represents an important technical advance for the community.

Reviewer 2 Comments for the Author...

This paper is much improved over the previous version, and the authors have address all of my comments and concerns. I particularly think the RNA-seq dataset, albeit at the bulk level (and

© 2022. Published by The Company of Biologists under the terms of the Creative Commons Attribution License (https://creativecommons.org/licenses/by/4.0/).

therefore difficult to distinguish between proportions of cell types rather than cellular identity), adds to the confidence that their in vitro embryos are mirroring in vivo development closely. My only remaining, minor comment would be that it could be useful to distinguish between DEG that are upregulated or downregulated between conditions (eg Fig 6A), as this might reveal some more insight - although I note that the data is available in table form if readers were inclined to examine it more closely.

We have included the number of upregulated genes in each condition for the different comparisons in Fig 6A.

Reviewer 3 Advance Summary and Potential Significance to Field...

The manuscript by Ramos-Ibeas and colleagues entitled “In vitro culture of ovine embryos up to gastrulating stages” provides an interesting way to get gastrulating ovine embryos from a novel in vitro culture system. They developed a new culture medium using N2/B27 supplements combined with ROCK inhibitor and activing A and used culture dish coated with agarose to avoid embryos' attachment to the dish. Using this system, they were able to produce gastrulating ovine embryos from an exclusively in vitro system.
The manuscript is of interest for a large panel of developmental biologist working on early embryonic development but also for those working on livestock reproduction and reproductive technology.

Reviewer 3 Comments for the Author...

The reviewer thanks the authors for all the improvements made in the manuscript and in the figures.
The revised version addressed the different concerns raised by the reviewer from the previous version.

One remaining problem is that the title is somehow misleading because in vitro cultured embryos only recapitulate some of the events corresponding to the gastrulating stages. The authors should therefore modify the title to make it closer to the reality of the model, something like "In vitro culture of ovine embryos up to stages recapitulating features of gastrulation".
We have modified the title accordingly to specify that we reached early gastrulating stages *in vitro*.

Similarly in Discussion, page 8 line 224-225 the conclusion has to be toned down.
We have toned down conclusions specifying that we reach early gastrulating stages *in vitro* in the discussion (line 224).
Minor points:

Fig.S1A and Fig/1C are redundant and provide the same information. Fig.S1A can be removed. We have removed Fig. S1A as suggested.

Page 5 line 143: Fig2C instead of Fig.2D
Thank you for the correction; this has been now modified.

Page 6, line 155: (E11, E12.5 and E14)
Thank you for the correction; this has been now modified.

Fig.S8 and lines 195 to 200: this result is of interest but is not really necessary and does not fit well in the text.
We agree and we have now removed this result.

© 2022. Published by The Company of Biologists under the terms of the Creative Commons Attribution License (https://creativecommons.org/licenses/by/4.0/).

Third decision letter

MS ID#: DEVELOP/2021/199743

MS TITLE: In vitro culture of ovine embryos up to early gastrulating stages.

AUTHORS: Priscila Ramos-Ibeas, Leopoldo Gonzalez-Brusi, Maria Torres-Used, Maria Jesus Cocero, Pilar Marigorta, Ramiro Alberio, and Pablo Bermejo-Alvarez
ARTICLE TYPE: Techniques and Resources Article

I am happy to tell you that your manuscript has been accepted for publication in Development, pending our standard ethics checks.

© 2022. Published by The Company of Biologists under the terms of the Creative Commons Attribution License (https://creativecommons.org/licenses/by/4.0/).
